# Enhancing Intelligent Shoes with Gait Analysis: A Review on the Spatiotemporal Estimation Techniques

**DOI:** 10.3390/s24247880

**Published:** 2024-12-10

**Authors:** Anna M. Joseph, Azadeh Kian, Rezaul Begg

**Affiliations:** Institute for Health and Sport, Victoria University, Melbourne, VIC 3000, Australia

**Keywords:** gait analysis, inertial measurement unit, spatiotemporal gait parameters, foot trajectory estimation, smart shoes, wearable sensors, portable, foot clearance, fall prevention, foot movement tracking

## Abstract

The continuous, automated monitoring of sensor-based data for walking capacity and mobility has expanded gait analysis applications beyond controlled laboratory settings to real-world, everyday environments facilitated by the development of portable, cost-efficient wearable sensors. In particular, the integration of Inertial Measurement Units (IMUs) into smart shoes has proven effective for capturing detailed foot movements and spatiotemporal gait characteristics. While IMUs enable accurate foot trajectory estimation through the double integration of acceleration data, challenges such as drift errors necessitate robust correction techniques to ensure reliable performance. This review analyzes current literature on shoe-based systems utilizing IMUs to estimate spatiotemporal gait parameters and foot trajectory characteristics, including foot–ground clearance. We explore the challenges and advancements in achieving accurate 3D foot trajectory estimation using IMUs in smart shoes and the application of advanced techniques like zero-velocity updates and error correction methods. These developments present significant opportunities for achieving reliable and efficient real-time gait assessment in everyday environments.

## 1. Introduction

Human gait, influenced by age, personality, and medical conditions, is crucial to an individual’s well-being. Alterations in gait can elevate the risk of falls and injuries, impacting quality of life. Gait analysis is the study of walking patterns and plays a crucial role in understanding biomechanical movements and identifying disorders or balance issues that deviate from the norm. With the emergence of smart technologies, gait analysis offers promising prospects for comprehensively assessing movement and mobility, aiding in sports activities, diagnosing medical conditions, and facilitating rehabilitation. By quantifying an individual’s risk of falling, gait analysis has become a valuable tool in fall prediction and prevention. Fall risk assessment systems monitor a person’s walking pattern over an extended period, predicting the likelihood of a fall occurrence, while fall detection systems serve as immediate assistive devices, promptly notifying caregivers or emergency services in the event of a fall [1]. Various factors, including stiffness, lack of coordination, impaired reflexes, reduced muscle strength and tone, as well as shorter step length and height, contribute to gait-related falls, highlighting the importance of comprehensive gait analysis in mitigating fall risks and enhancing overall safety [2].

Conventionally, gait analysis was carried out subjectively through visual observations but with technological advancements; motion analysis systems proved to be more efficient and accurate in analyzing the gait [3,4]. Motion analysis systems, particularly those utilizing motion capture systems synchronized with force platforms, have become the gold standard for accurate and reliable gait analysis [5]. These systems are highly accurate and allow for comprehensive data collection in controlled environments, facilitating detailed spatial and temporal movement analysis. Optical markers positioned on the heel and toe enable the measurement of their respective trajectories, facilitating the identification of gait events and spatiotemporal parameters [6]. Despite their high accuracy, these systems demand substantial expenses and expertise, limiting mobility and confining analysis to laboratory settings. Additionally, extended setup times, postprocessing requirements, and restrictions related to the movement area and gait cycles for the observed subject/patient have been noted. To overcome limitations associated with current methods, researchers have explored wearable sensor technologies for gait analysis [7,8]. These technologies offer portability, enabling real-world data capture and enhancing subject mobility. Wearable gait analysis systems have evolved rapidly, becoming more versatile, portable, inexpensive, and robust through the integration of sensors, signal processing, and advanced gait detection algorithms. Despite these advancements, wearable systems may sacrifice some precision and comprehensive data coverage compared to traditional motion analysis systems, but they provide significant advantages in terms of accessibility and usability outside laboratory settings. The choice between these methods hinges on the specific research objectives, as motion capture systems excel in detailed and accurate analysis in controlled environments, while wearable sensors thrive in real-world scenarios and ambulatory settings, focusing on mobility and practicality.

Recent years have seen wireless, battery-powered, real-time Inertial Measurement Units (IMUs), as an alternative to gold standard solutions, with many researchers focusing on developing robust methods to ensure the systems can be used by patients in unsupervised settings both indoors and outdoors [9,10]. As research progresses, IMUs have become smaller, lighter, more accurate, and more cost-effective, as well as more energy-efficient, measuring single or multi-point motion trajectories of single or multiple body segments of the subject during gait, and they have become indispensable in several fields of research for performing complex motion analysis [11,12]. These units can measure the motion of the body segment to which they are attached, making them valuable in gait analysis for assessing motion and joint angles [13]. Gait detection algorithms based on IMU sensors encompass various methodologies, including rule-based methods, signal processing techniques, or machine learning-based methods. Rule-based methods set a predefined criteria like threshold for the acceleration or angular velocity to detect heel strikes or toe off. Signal-processing techniques analyze and manipulate raw sensor data and apply filters like low -pass filters to remove noise and Kalman filter, extended Kalman filter, and complementary filter to estimate the positions and orientation [13,14,15].

For the estimation of gait events and spatiotemporal gait parameters, placing the IMU sensors on the foot is recognized as one of the most effective methods [16,17]. This optimal placement allows the precise measurement of parameters such as stride length, gait speed, cadence, swing width, and foot clearance. These parameters are vital for assessing walking performance, particularly in older adults, with gait speed being a key indicator of overall health and functional ability [18]. Insufficient toe clearance during walking can lead to tripping over small obstacles or uneven surfaces, making it a significant factor in evaluating fall risk and overall mobility safety in the elderly population [19,20]. Smart shoe systems incorporating embedded sensors in shoes for gait analysis represent a sophisticated approach to capture and analyze various aspects of an individual’s gait pattern, especially foot clearance and trajectory.

Theoretically, integrating acceleration data from an accelerometer twice yields positions, and integrating angular velocity data from a gyroscope provides the relative change in attitude [21]. However, in real-world applications, these sensors often experience noise, and even small errors can result in significant drift when the raw measurements are integrated. The drift in gyroscopes and accelerometers leads to a linearly growing error, particularly in position estimation, where the error can increase to meters within a few seconds and reach hundreds of meters within a minute. This makes it impractical to rely solely on double integration for accurate position estimation [21]. Researchers have implemented additional measures to acquire domain knowledge and eliminate drift by leveraging the periodic nature of human gait [21,22,23,24,25]. The objective of this review is to investigate existing literature on shoe-based systems that utilize IMUs for estimating spatiotemporal gait parameters with a specific focus on estimating foot clearance and foot trajectory. The current article aims to analyze and understand the methodologies employed to mitigate drift and improve accuracy in such systems.

A search was conducted using Scopus to explore literature on foot clearance, spatiotemporal gait estimation, and foot trajectory estimation with IMUs in footwear. Keywords such as “foot clearance estimation with IMU” and “foot trajectory estimation” were utilized. Abstracts were screened to select studies specifically involving IMUs integrated into shoes or feet. The search was further refined to include only studies that exclusively used IMUs, focusing on either spatiotemporal parameters or foot trajectory analysis. This refined search resulted in a final selection of 14 papers aligned with the review’s objectives.

A selection of the state-of-the-art shoe systems developed for gait analysis, discussed in the reviewed articles, are shown in Table 1 for illustration purposes.

## 2. Human Gait Analysis

**Figure 1:**
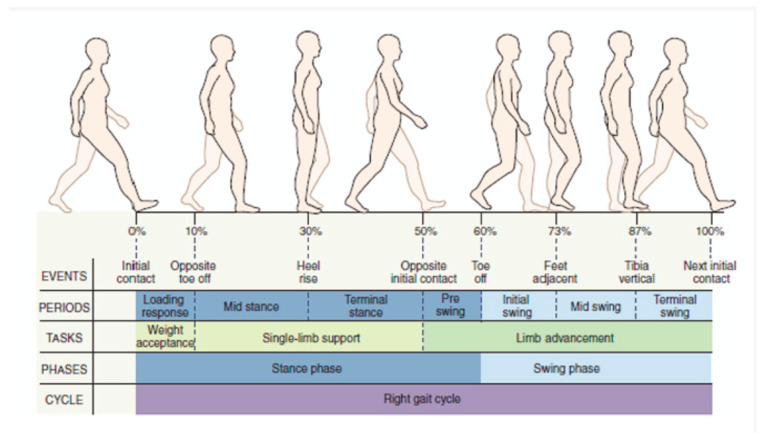
Illustration of different gait events during a gait cycle. Reprinted from [32].

Gait analysis is the comprehensive evaluation of human walking. Walking is an oscillating process with the cyclic rotation of the human limbs or body segments and the measurement, description and assessment of the human walking pattern by segmenting into phases, estimating the kinetic and kinematic parameters, and quantitatively evaluating the musculoskeletal functions [8]. A gait cycle, defined as the time period between two successive occurrences of one of the repetitive events of walking, is approximately 1 s in duration and is divided into two main phases: stance phase and swing phase [29,30]. The stance phase starts with the foot strike on ground and ends when it leaves the ground, constituting 60% of the gait cycle, while the swing phase is where the leg swings through and is not in contact with the ground, accounting for the remaining 40% of the gait cycle [31]. The gait cycle is further divided into eight sub-phases: initial contact, loading response, mid-stance, terminal stance, pre-swing, initial swing, mid-swing and terminal swing [29]. It is the sequential combination of these phases that enables the limb to perform the three basic tasks: weight acceptance, single-limb support and limb advancement. Kharb, Saini, Jain and Dhiman [30] described each of these phases as below:

- Initial Contact: The heel just touches the floor, and the joint postures determine the limb’s loading response pattern.
- Loading Response: Body weight transfers to the forward limb, marking the start of the double-stance phase until the other foot lifts.
- Mid-Stance: The first half of single limb support, beginning when the other foot lifts and bodyweight aligned over the forefoot.
- Terminal Stance: Completes the single-limb support, beginning with heel rise ending with opposite foot striking the ground.
- Pre-Swing: The second double-stance interval in the gait cycle, starting with the opposite foot’s initial contact and ends with the ipsilateral toe-off, thus positioning the limb for swing.
- Initial Swing: Begins when the foot lifts off the ground and ends when the foot is opposite to the stance foot, accounting for one third of the swing period.
- Mid-Swing: The second swing phase, starting when the foot is opposite the stance foot and ending when the tibia is vertical.
- Terminal Swing: Completes the limb advancement, beginning with a vertical tibia and ends when foot strikes the floor (see Figure 1).

Basic gait analysis measures the spatiotemporal parameters like walking velocity, stride length, step length, stride time, step time stance and swing time and cadence. Stride length is the distance between successive placements of the same foot. Step length is the distance between two subsequent heel strikes of the opposite limb and two steps constitute a stride. Cadence is the number of steps per minute [33]. Parameters such as stride length and walking speed, which change with age, have been identified as factors related to the increased risk of falling in the elderly [34,35]. Additionally, these gait characteristics can serve as diagnostic parameters for various adult diseases, leading to a significant focus among clinical researchers on developing technologies for measuring them. Gait parameters vary with age, and Table 2 presents the healthy gait parameters for different age ranges as reported in the literature.

Motion analysis systems, particularly those utilizing motion capture systems with force platforms, have become the gold standard for accurate and reliable gait analysis [5]. These systems typically consist of cameras and markers placed on specific body parts to capture and track movement. Cameras record the movement of reflective or active markers, capturing their positions in space. The data collected by these markers are then processed to analyze the movement of the body in three-dimensional space. Various motion capture systems such as Vicon [37,38], OptiTrack [39] and Qualisys [40] have been employed to collect gait data. Qualisys and Vicon are based on a passive marker setup (reflective) coated with a retroreflective material and tracked with the infrared red light from the motion cameras. OptiTrack is based on an active marker setup generating infrared light, thus enabling the motion cameras to track the markers. A number of analysis techniques have been implemented to study the pattern of the gait [41,42], understand the gait intention such as standing still, walking [43,44] and predict the risk of tripping [45,46,47]. Motion capture systems are pivotal in understanding gait biomechanics, aiding in research, clinical diagnosis, and the development of treatments for various gait-related conditions. They offer in-depth, quantitative insights into human movement, helping improve treatment strategies and enhancing the overall quality of gait analysis. However, it is important to note that gait analysis utilizing indoor motion capture systems is confined to laboratory environments and can involve significant expenses. The restrictive nature of this setup limits its usage to controlled indoor spaces, excluding real-world or outdoor settings where natural locomotion occurs. With increased versatility, gait detection devices have progressed rapidly in the process of making portable, inexpensive, and robust solutions, embedding sensors, incorporating signal processing and gait detection algorithms.

## 3. Wearable Sensors for Gait Analysis

Wearable sensors in gait analysis have emerged as a transformative tool, heralding a new era in comprehending human mobility [7,8]. Their compact, user-friendly design has unlocked unprecedented possibilities in understanding gait patterns, diagnosing movement disorders, and enhancing rehabilitation techniques. The rapid and continuous evolution of smaller, more robust, and efficient sensors has notably improved the objective and quantitative assessment of gait characteristics while offering increased convenience [7,48].

The diverse array of wearable sensor types allows for multi-dimensional analysis, offering comprehensive insights into the intricacies of gait. These sensors facilitate real-time data collection, continually recording movement metrics as users perform their daily activities. Such real-time monitoring is particularly valuable in clinical settings, providing instant feedback for clinicians, researchers, and rehabilitation specialists. The capacity to adjust interventions or treatments based on real-time data substantially enhances the quality of patient care and research outcomes. Emphasizing sensor placement and type selection is crucial, as the positioning of wearable sensors significantly impacts the accuracy of recognizing body motions, with most studies indicating that recognizing more complex activities necessitates multiple sensors in various locations [49].

These sensors encompass various types, each with its own set of advantages and limitations. Inertial Measurement Units (IMUs), integrating accelerometers, gyroscopes, and sometimes magnetometers, offer cost-effective and portable solutions [41,50]. IMUs excel in collecting orientation and acceleration data, making them ideal for studying movement patterns and gait dynamics. Accelerometers, by measuring changes in acceleration, play a pivotal role in gait analysis, capturing step counts and movement patterns. Their portability and accessibility make them useful in tracking everyday activities, yet their precision can be affected by calibration and drift. Additionally, accelerometers are noisy, subject to the influence of gravity, and sensitive to both position and orientation [51]. Gyroscopes, on the other hand, track orientation and rotation, offering insights into angular motion during gait. They find applications in analyzing human movement but face challenges related to power consumption. Magnetometers are sensitive to magnetic fields, and they complement gyroscopes and accelerometers by contributing to navigation and aiding in gait analysis. However, they are prone to interference. Pressure-sensitive insoles or in-shoe force sensors provide insights into plantar pressure distribution during walking, aiding in assessing foot biomechanics and gait patterns. Nevertheless, these sensors may be limited in their ability to collect comprehensive motion data beyond foot pressure [51]. Electromyography (EMG) sensors record muscle activity, aiding in muscle involvement analysis during gait. While they offer crucial insights into muscle function early, EMG sensors can be complex to set up and might be influenced by external factors like skin artifacts, cross-talk, and movement artifacts, requiring extensive preprocessing [51]. Goniometers measure joint angles during gait, enabling precise kinematic analysis. Their applications in diagnosing gait abnormalities and assessing joint movement are invaluable, yet their reliance on accurate joint placement and potential interference remains a challenge [52]. Each type of sensor has its own strengths and limitations, offering a spectrum of information for gait analysis, and the selection depends on the specific research needs and practical considerations. Table 3 shows the major features, advantages and disadvantages of commonly used wearable sensors used for gait analysis.

Reviews analyzing wearable technology for gait analysis consistently indicate that IMUs are widely employed in studies focusing on walking and running analysis. A systematic review involving 131 articles on wearable technology used for gait analysis showed that most studies used IMU sensors, with 14 solely using accelerometer capability, and 20 using accelerometers and gyroscopes [7]. Prasanth, Caban, Keller, Courtine, Ijspeert, Vallery and von Zitzewitz [51] focused on analyzing wearable sensors used for real-time gait detection and highlighted the widespread use of IMUs for gait analysis. Additionally, this study noted the use of FSR sensors for ground-truth validation purposes [51]. Embedding force sensors in shoe insoles has been shown to improve timing accuracy [54]. A combination of IMUs along with footswitches or force sensors in insoles for gait events detection is utilized in numerous studies [7,51,55]. While these methods offer superior detection performance, they require additional hardware, making them more expensive than IMU-only solutions and less convenient for patients due to the need to wear specific shoes and insoles. Over time and with continued use, the sensitivity and accuracy of FSRs can degrade, leading to diminished performance and requiring frequent maintenance or replacement. Due to their ease of attachment to the user’s shoe without requiring specialized footwear, ensuring user comfort, and minimizing sensor count, many studies are prioritizing IMU-only solutions to accurately estimate foot trajectory.

## 4. Inertial Measurement Units

IMU sensors typically include accelerometers, gyroscopes, and magnetometers, and they are being increasingly used to collect objective gait data measuring the acceleration, angular velocity and magnetic field related to the motion of objects where the sensors are fixed [56]. Their advantages—such as high sampling rates, affordability, portability, ease of use, suitability for outdoor settings, and simpler calibration compared to motion capture (Mocap) systems—make IMUs an attractive alternative to traditional motion capture systems. Both commercially available and researcher-developed IMU systems have been utilized to measure human movement across various domains, including sports and clinical settings [11,13,57]. Attachable to almost any body part or shoes, IMUs allow researchers and clinicians to capture detailed information about gait patterns and dynamics in real-world environments.

The data collected from these sensors can be processed through feature detection or more advanced techniques that combine inputs from multiple sensors, allowing for the estimation of various parameters. Temporal parameters involve identifying specific time points or intervals, such as the start and end of movements, contact events, and stride durations. Kinematic parameters encompass the estimation of both angular and linear positions, velocities, and accelerations, including the 3D orientation of a MIMU relative to an inertial frame, which is essential for understanding body segment orientations and joint kinematics. Dynamic parameters, like forces, moments, and powers, provide insight into the forces involved in movement but rely heavily on accurate kinematic data [58]. These parameters provide valuable insights into various movements and can be accurately measured when sensors are positioned correctly on the body.

**Figure 2:**
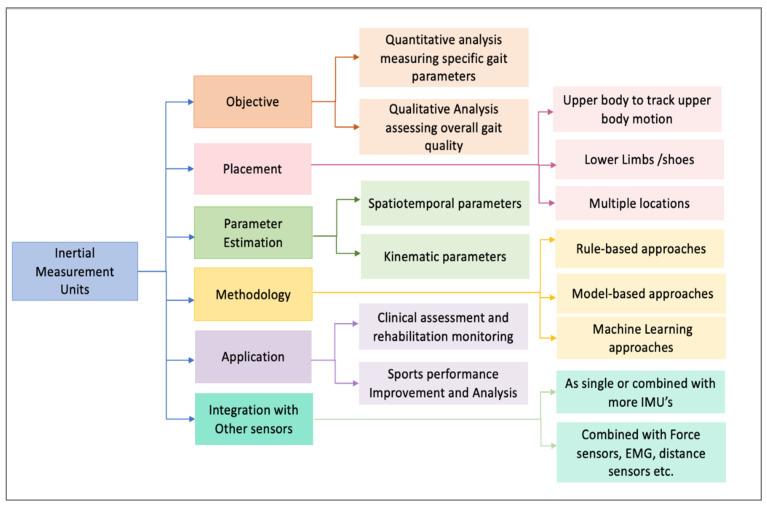
Taxonomy of IMUs for gait analysis [48].

Common anatomical landmarks for sensor placement vary depending on whether the focus is on upper or lower limb motion. For upper limb motion analysis, sensors are typically attached to the trunk, back, scapula, lumbar region, upper arm, forearm, and hand, while for lower limb motion, they are placed on the trunk, lower back, sacrum, hip, pelvis, thigh, shank, and feet [13]. The development of wireless, battery-powered IMUs that can be easily attached to these body parts allows for real-time, comprehensive gait analysis in both indoor and outdoor, unsupervised settings. Figure 2 illustrates a taxonomy that categorizes the use of IMUs in gait analysis, providing a structured understanding of their diverse applications and methodologies.

IMUs are widely used in various applications, including clinical gait analysis and sports performance analysis. In clinical settings, IMUs help assess gait abnormalities, monitor rehabilitation progress, and inform treatment decisions [59,60]. In sports, IMUs are used to analyze movement patterns, improve performance, and prevent injuries by identifying biomechanical inefficiencies [58,61].

Gait analysis with IMUs can be categorized as having two objectives: quantitative analysis and qualitative analysis. Quantitative analysis involves measuring numerical gait parameters, such as stride length and step time, for analysis, allowing researchers and clinicians to obtain detailed information about an individual’s gait pattern and dynamics [62,63]. On the other hand, qualitative analysis focuses on assessing overall gait quality and pattern based on IMU data, offering a more holistic view of gait performance [57,64,65,66]. Spatiotemporal parameters, such as stride length, step time, and walking speed, are estimated to characterize an individual’s gait pattern and dynamics [67,68]. Kinematic parameters, including joint angles and segment orientations during gait, provide insights into joint motion and coordination [68,69].

Gait analysis using IMUs (Inertial Measurement Units) can be approached through either rule-based or machine learning methods [57]. Rule-based approaches rely on predefined algorithms and heuristics, such as detecting peaks [25,70], identifying zero-crossing points [71], and applying thresholds [9,22,72,73] to recognize gait events. Although these methods are typically straightforward, computationally efficient, and easy to understand, they may struggle with varying walking conditions due to their sensitivity to noise and limited adaptability. On the other hand, machine learning approaches are designed to learn and recognize specific features within the periodic patterns of gait phases and transitions, making them adaptable to a wide range of gait conditions [28,74].

The accurate placement of Inertial Measurement Unit (IMU) sensors is critical for capturing reliable gait data, depending on the specific analysis objectives. These sensors can be strategically placed on specific body parts to capture detailed information about different aspects of movement during gait [51]. They are often placed on the lower limbs, including the feet, shanks, and thighs, to capture foot motion, leg movements, and joint angles [75]. They can also be attached to the trunk, including the pelvis and lower back, to monitor trunk motion and posture during gait [76], or on the upper limbs, including the arms and hands, to observe arm motion and coordination [11]. Comparisons of the temporal gait parameters across different IMU placements such as on the trunk, shank and foot revealed foot IMU data to be the more accurate among the three [17]. Furthermore, an evaluation of 17 algorithms based on the IMU data and sensor placement confirmed that foot-based algorithms for detecting heel strikes and toe-offs offer superior accuracy and repeatability [16]. Foot-mounted IMUs allow for high spatial accuracy in gait analysis by enabling techniques such as zero-velocity updates, which can be translated into detailed spatiotemporal gait metrics [21].

The Minimum Toe Clearance (MTC) is the shortest distance between the toe and the ground observed during the mid-swing phase of the gait cycle, which is typically occurring when the foot achieves peak velocity. A combination of high forward velocity, low MTC, and single-foot support can significantly increase the risk of tripping and falling, especially in individuals with shuffling gait or restricted lower limb movement. This risk is particularly pronounced in people with neuromuscular conditions, muscle weakness, or other impairments that affect gait stability and control [24]. Due to the predictive value of low MTC in evaluating tripping risks, there has been a significant amount of research focused on estimating foot clearance or trajectory and spatiotemporal parameters with shoe-based systems [28,37]. However, processing the data to estimate the 3D position evolution of the attached limb is still challenging due to the error characteristics of the underlying sensors.

## 5. Smart Shoes for Gait Analysis

Embedding sensors on or inside shoes is a promising area, enhancing user acceptance by seamlessly incorporating technology into a commonly worn item. The integration of such cutting-edge technology not only enhances our understanding of gait dynamics [36,54,77] but also extends its applications to posture and human activity recognition [78,79], detecting gait abnormalities [54,80], energy expenditure estimation, biofeedback, navigation, and fall risk assessment [1]. Smart shoes capable of fall detection and prediction have been used in fall risk assessment and prevention. The existing fall prevention systems monitor the individual’s gait over prolonged time and predict risk of falling, while fall detection systems alert when a fall event has occurred [1]. By continuously monitoring foot movements and ground interactions, smart shoes provide valuable insights into parameters such as step length, cadence, gait cycle, and even the timing of specific gait events like heel strikes and toe-offs [54].

Several advantages of using smart shoes as smart devices, for the purpose of mobility assessment, have been identified in research:

- Smart shoes have a predefined, rigid sensor position on the foot, providing accurate, repeatable and flexible biomechanical analysis.
- Smart shoes can be used to monitor gait, which is a highly stereotyped movement that enables the automated assessment of functional biomechanics; and
- Smart shoes enable a non-obtrusive and non-stigmatizing integration of technology, ultimately improving user acceptance and long-term adherence [81].

**Figure 3:**
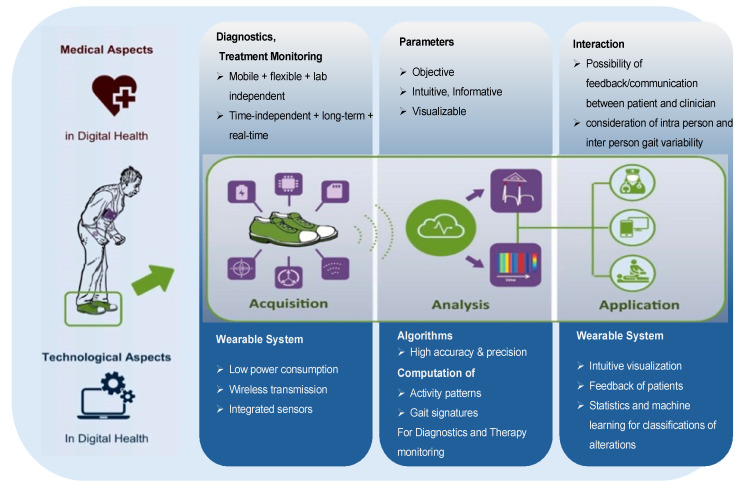
Illustration of the medical and technological requirements for Internet of Health for gait monitoring. Adapted from [81].

Gait assessment with smart shoes necessitates a comprehensive data acquisition system that not only collects sensor data but also efficiently manages power consumption and ensures the reliability of data generation, storage, and analysis [81] (see Figure 3).

**Figure 4:**
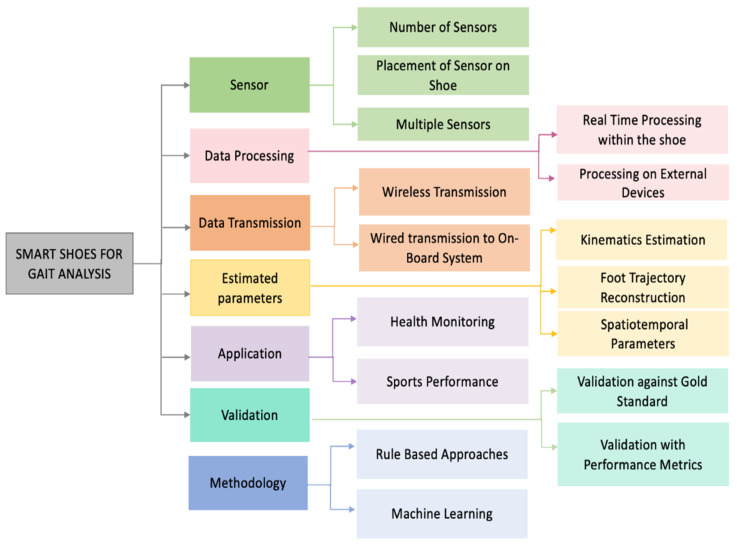
Taxonomy of smart shoe for gait analysis illustrating existing research categories [1,10,15,22,25,26,27,55,70,73,83].

Efficient power management is a critical aspect of smart shoe design to ensure the system can continuously collect relevant gait data without frequent recharging [81]. Smart shoes integrate various wearable sensors that capture detailed foot movement and pressure data, which are essential for identifying key gait events and patterns [1]. Optimizing energy consumption through low-power sensors, energy-efficient algorithms, and intelligent power management strategies extends battery life, making these devices more user-friendly [1,81]. Efficient data storage solutions, such as low-power memory modules, enable the system to record and retain extensive gait data for later analysis. The integration of wireless transmission capabilities allows for real-time data access and remote monitoring, further enhancing the functionality and user experience of smart shoes [81]. Reliable data generation and advanced processing techniques, such as filtering and machine learning algorithms, are essential for extracting meaningful and actionable insights from raw sensor data [31,82]. Efficient algorithms process the collected data to identify key gait events and patterns accurately. A taxonomy of smart shoes systems used for gait analysis is shown in Figure 4.

Placing IMU sensors on the shoe has proven effective for estimating foot trajectories and determining spatiotemporal characteristics [25,83]. This placement allows the sensors to capture essential data on foot movement and positioning, providing valuable insights into gait patterns and aiding in the assessment of walking performance. Foot trajectories are typically computed through the double integration of acceleration data, necessitating precise sensor orientation to differentiate linear acceleration from gravitational forces [25,70]. Robust drift correction techniques are essential to address errors that accumulate during this integration process [9,25,70]. Accurate foot trajectory and clearance estimation using smart shoes with IMUs is important for diagnosing gait abnormalities, preventing falls, and enhancing safety for users [15,70,72]. Understanding these technologies is required for delivering real-time feedback and achieving more reliable gait analysis. The next section will explore the methodologies for foot trajectory and clearance estimation with a focus on IMU integration in smart shoes.

## 6. IMU Applications on Shoe: Position, Trajectory, and Spatiotemporal Estimation

Studies that focus on the estimation of spatiotemporal parameters using only data from shoe-mounted IMUs primarily serve three key purposes: position estimation, foot trajectory reconstruction, and spatiotemporal parameter analysis.

- Position Estimation: Determining the location of a person relative to a fixed frame of reference. It involves estimating the movements and spatial coordinates of the person in a given environment or scenario [84].
- Foot Trajectory Reconstruction: This involves reconstructing the path traced by the foot in space over time [72,85].
- Spatiotemporal Estimation: Encompasses both spatial (distance, position) and temporal (time-related) aspects of human movement using IMU data. Step length, step width, along with parameters such as stride duration, cadence, stance phase, swing phase, and gait velocity, collectively fall under spatiotemporal estimation [10,21,22,26,27].

The position estimation helps to understand the spatial coordinates of the person over time in relation to a predefined reference frame. The primary goal of this research is to understand the foot trajectory movement in three-dimensional space. This involves detecting specific events like toe-off and heel-strike without requiring a known starting point. By analyzing data from the IMUs, the spatiotemporal parameters such as step length, step width, gait cycle duration and foot clearance can be derived. Thus, while position estimation is concerned with the overall spatial coordinates relative to a fixed frame, our focus is on detailed foot motion analysis and event detection using IMU data that facilitate the calculation of key spatiotemporal parameters critical for understanding human movement dynamics. In this paper, the term ‘position estimation’ refers to the calculation of displacement or distance based on acceleration data.

In the context of estimating the spatiotemporal parameters, IMUs (Inertial Measurement Units) are commonly employed in a two-stage approach. To estimate the foot trajectory, researchers have employed a two-step approach. Initially, they isolate the gait events, and subsequently, they apply spatiotemporal estimation techniques [9,21]. Gait event detection from IMU data involves utilizing either acceleration [70,73], angular velocity [21,22,26], or a combination of both [9]. In spatiotemporal estimation, the primary procedure involves translating acceleration data from the sensor coordinate system to the Earth coordinate system [15,21]. Following this translation, the double integration of acceleration data is used to calculate or estimate the position [9,10,15,22,70,72,83]. To mitigate drift error accumulated during integration, the zero-velocity update (ZUPT) assumption is applied, considering the foot stationary during the stance phase, hence velocity being zero [9,21,26,72].

Although the process of estimating spatiotemporal parameters appears straightforward, multiple sources of errors complicate the process, making it challenging to obtain accurate values. Inaccuracies can arise from sensor noise and drift in accelerometers and gyroscopes, which accumulate over time, leading to errors in the calculated position and velocity [15]. Misalignment of the IMU on the body or poor calibration can result in erroneous measurements, affecting the reliability of spatiotemporal parameters [9]. When estimating orientation, the integration of gyroscope readings leads to accumulated drift, and this drift subsequent affects velocity estimation, where accelerations are transformed into the global coordinate system and integrated to obtain linear velocity [21,72]. Additionally, magnetometer equipped IMUs are susceptible to distortions from external magnetic fields, complicating orientation estimation [15,51]. Natural variability in gait patterns, along with incorrect assumptions about initial conditions, can propagate errors, and numerical integration further amplifies these inaccuracies over time, causing small errors to accumulate and resulting in significant drift in velocity and position estimations [15].

Researchers have conducted numerous studies and developed various techniques to mitigate these errors. Several research groups have reported that the use of drifting methods can provide accurate estimation of stride length during walking and running across a range of speeds [9,15,26,73]. Therefore, the rest of the section will discuss the development of a shoe-based system utilizing IMUs for estimation and the drift correction techniques employed for analyzing the 3D foot trajectory and foot clearance.

A literature review was conducted to examine techniques employed by researchers for estimating foot trajectory, foot clearance, and related parameters. Table 4 summarizes findings from analyzed studies, emphasizing sensor placement, methodologies, and evaluation techniques investigated across various research domains. Additionally, Table 5 provides an overview of the significance and limitations of each system.

In studies focusing on estimating spatiotemporal parameters using IMUs, researchers typically opt for one of two configurations: placing one IMU on each foot [9,10,15,22,70,72,83] or utilizing a single IMU on one foot [25,26,27,73,85,86]. The placement on both feet allows for the measurement of movement and orientation data from both feet simultaneously, providing comprehensive spatiotemporal information. The dorsum (top) of the foot emerges as the predominant location for sensor attachment in these investigations, as noted in several sources [9,15,21,25,85]. However, in some cases, alternative placements are explored for specific reasons such as comfort or technical requirements [15,83]. For instance, in [27], the IMU is positioned on the arch of the shoe. This placement was chosen to enhance comfort, which necessitated designing a dedicated insole with a specific area to accommodate the sensor securely.

Researchers have utilized inertial measurement modules equipped with built-in CPUs or microcontrollers, memory, Bluetooth, and batteries, enabling independent operation of the IMU [25,27,28,85]. Data can either be logged onto a local flash memory [26] or streamed wirelessly via Bluetooth to a mobile device or PC [27]. Additional user-friendly features include wireless charging on customized platforms and a graphical user interface (GUI) for initiating or terminating recordings, the real-time monitoring and visualization of walking data, immediate analysis of gait data post-walking, and autonomous generation of performance feedback reports [26].

### 6.1. Data Processing

**Figure 5:**
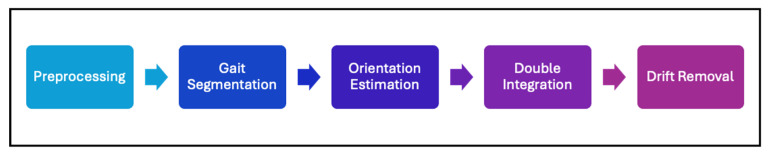
Illustration of steps for estimating foot trajectory and spatiotemporal parameters based on literature analysis [9,10,15,22,25,26,27,70,72,73,83,85,86].

The processing of gait data is categorized into either offline analysis, where data are collected and processed in batches, sometimes in short intervals or at the end of the entire trial [9], or real-time analysis, which involves analyzing data during the walking task itself, providing immediate feedback [26,27,86]. Research studies often use offline analysis for comprehensive and detailed investigations, while real-time analysis is valuable for applications requiring immediate feedback or intervention, such as rehabilitation or wearable technologies for daily use. Estimating foot trajectories from IMU data typically involves gait segmentation, orientation estimation, velocity estimation, zero-velocity updates with drift correction techniques, and position estimation, as illustrated in Figure 5 based on our review of the literature.

#### 6.1.1. Preprocessing

The raw sensor data from IMUs typically undergoes several crucial preprocessing steps to ensure accuracy and reliability in subsequent analyses. Calibration is a critical initial step, involving systematic adjustments to correct sensor biases and align output with established standards [15]. This process may utilize specialized equipment or innovative methods such as 3D-printed structures like an icosahedron, which provide controlled environments for precise calibration without traditional devices [88]. Benoussaad, Sijobert, Mombaur and Azevedo Coste [15] calibrated the IMU by placing it in over 20 different random orientations and measuring the acceleration vectors under static conditions, ensuring the magnitude of the acceleration vector equals the gravity vector (9.81 m/s^2^). Meanwhile, Nguyen and La [86] utilized an L-shaped calibration frame to align the IMU’s coordinate system with a standardized reference frame ensuring consistent and accurate orientation data. In addition to traditional calibration methods, some studies have explored calibration-free approaches for assessing gait using IMU sensors, eliminating the need for extensive calibration procedures [9,10]. Calibration-free techniques eliminate the need for IMUs to be aligned with specific anatomical axes or to undergo a predefined calibration process for orientation adjustment. Instead, they leverage arbitrary sensor placement and non-causal, batch signal processing methods that account for variability and misalignment, enabling accurate gait measurements without explicit calibration [9]. This approach offers significant advantages in terms of simplicity, reduced setup time, and greater flexibility in real-world applications, making it especially useful for diverse and dynamic environments. Following calibration, filtering is applied to the data to eliminate high-frequency noise that could distort the signals [15,25]. For example, Benoussaad, Sijobert, Mombaur and Azevedo Coste [15] employed a Butterworth filter on accelerometer and gyroscope data to effectively reduce high-frequency components. Filtering ensures that subsequent analyses are based on accurate representations of intended movements.

Furthermore, proper sampling techniques are indeed crucial in digital signal processing, particularly when dealing with IMU data, to prevent aliasing effects [22]. Aliasing occurs when high-frequency components of a signal are incorrectly represented as lower frequencies due to insufficient sampling rates, distorting the digital data representation. To address this issue, an anti-aliasing filter is employed before digital sampling to attenuate frequencies above the Nyquist frequency sampled and mathematically transformed into the frequency domain by the fast Fourier transform [26].

These preprocessing steps—calibration, filtering, and sampling—are integral to preparing IMU data for accurate estimation of foot orientation, movement dynamics, and spatiotemporal parameters [22,25].

#### 6.1.2. Gait Segmentation

In a gait cycle, the most prominent movement is the rotation of the foot around the mediolateral axis comparing to supination/pronation and inversion/eversion. Key gait events such as heel strike (HS), foot flat (FF), and toe-off (TO) can be detected by analyzing the rotational movement aligned with the mediolateral axis of the foot [26]. In a normal gait cycle, the foot starts flat on the ground, rotates forward with the toe as the contact point while the heel lifts and rotation speeds up, then slows down as the toe leaves the ground, reverses as the foot moves forward and the toe elevation exceeds the heel, reverses again after the heel strike, and ends with the toe lowering down using the heel as the contact point until the foot is flat again [21].

Existing methods often vary in the gait events they detect, typically focusing on distinguishing stance and swing phases but sometimes also identifying additional events like mid-swing. Commonly, many researchers identify four specific events: initial contact, full contact, heel rise, and toe-off, though the terminology may vary [9]. Some studies also include the detection of the flat foot phase or mid-stance for drift removal. Mid-stance occurs when the entire foot is in contact with the ground during the stance phase of walking. During this phase, characterized by the complete contact of the foot’s sole with the ground, the Euclidean norm of the accelerometer readings tends to approximate 9.81 m/s^2^, reflecting the influence of gravity and angular velocity near to zero [85]. Simultaneously, the norm of the gyroscope readings typically approaches zero, indicating minimal rotational movement of the foot around its axes during this stationary phase of the gait cycle [9].

**Figure 6:**
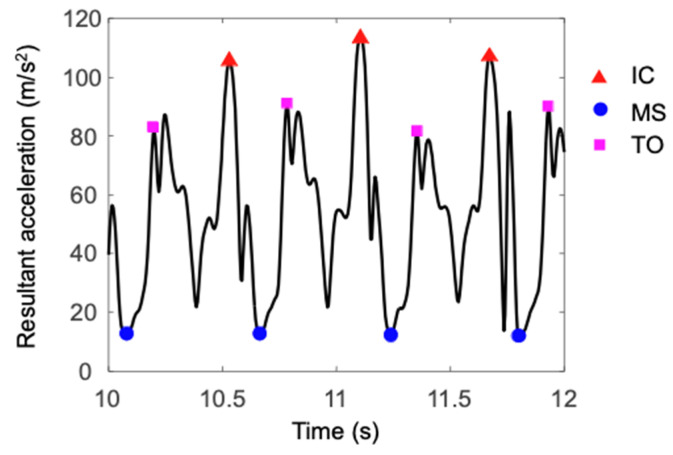
Visualization of the gait segmentation based on the peak of acceleration signals. IC: initial contact, MS: mid-stance, TO: toe-off. Reprinted from [70].

Gait event detection from IMU data involves utilizing either acceleration, angular velocity, or a combination of both. Threshold-based methods and heuristic peak detection techniques are commonly used to identify these key events in the gait cycle. The acceleration and angular velocity data shows specific peaks at the start and end of stance phase, which are indicative of gait events heel strikes and toe off. A prominent peak typically corresponds to moment when the heel makes initial contact with the ground and another distinct peak often signifies when the toes lift off from the ground, preparing for the swing phase (see Figure 6). These peaks in both acceleration and angular velocity data serve as clear markers for detecting and understanding the timing and dynamics of key events in walking or running. Threshold-based methods set specific threshold values for peaks movement data, and when the value surpasses these thresholds, they trigger the detection of specific events such as heel strikes and toe-offs [21,22]. Heuristic peak detection uses rules or algorithms to identify characteristic patterns in movement data corresponding to heel strikes and toe-offs, incorporating criteria beyond simple thresholding, such as peak shape, duration, and context within the gait cycle. Suzuki, Hahn and Enomoto [70] defined initial contact as the first instant of peak resultant acceleration and detected toe-off as the instant of the first peak of resultant acceleration in the time range of 0.1 s to 0.4 s following initial contact. Kitagawa and Ogihara [85] detected flat foot as the period during which the magnitude of the angular velocity vector remains below 25°/s for at least 0.1 s, while Benoussaad, Sijobert, Mombaur and Azevedo Coste [15] detected it as the minimum of angular velocity in each stride cycle.

Unlike fixed threshold methods that use a constant threshold for detection, some studies employ adaptive thresholding, which dynamically adjusts threshold values based on the characteristics of the data being analyzed [87]. Laidig, Jocham, Guggenberger, Adamer, Fischer and Seel [9] proposed an automatic adaptive thresholding method that dynamically determines the thresholds for each trial based on the measured data.

For the real-time detection of the current gait phase with immediate feedback, a threshold-based approach was employed on the foot sole angle, which was coupled with a temporal window. This method monitors changes in the foot sole angle over a defined time interval to accurately identify transitions between stance and swing phases during walking [27]. Guimarães, Sousa and Correia [87] utilize angular rate data from IMUs along with signal processing techniques such as energy calculation, adaptive thresholding, and interval refinement to effectively detect zero-velocity intervals (ZVIs) during walking, ensuring reliable detection across varied walking speeds and conditions, thereby enhancing the accuracy and dependability of motion analysis employing IMUs. To detect gait events without relying on sensor orientation, acceleration magnitude and the vertical component of acceleration in global coordinates can be used. This approach avoids the need to precisely align the sensor on the body, simplifying gait event detection across different sensor placements [87].

Gait information can be obtained by analyzing the energy fluctuation across all three axes of the accelerometer [73]. The acceleration energy varies with foot movements with it being high when the foot is in the air and decreasing to zero when the foot contacts the floor. The energy fluctuation from all three axes can be calculated from the acceleration amplitude and utilized for gait segmentation by applying an energy threshold [73].

#### 6.1.3. Orientation Estimation

Orientation information provides the spatial orientation and movement dynamics necessary to accurately model and predict the foot movement through space and time. It involves understanding how the foot rotates and tilts relative to a fixed reference frame, facilitating the determination of its position and alignment in three-dimensional space. Near or on the Earth’s surface, orientation is typically described using two coordinate systems: the earth-fixed coordinate system (or global coordinate system), aligned with the local north, east, and down directions (NED), and the body-fixed coordinate system, which is based on the orientation of the body to which the sensor is attached [56]. In global coordinate systems, the vertical axis is aligned with the direction of gravity, pointing toward the center of the Earth [89].

Research in orientation estimation often utilizes the direction cosine matrix, Euler angles or quaternions to represent the orientation. The direction cosine matrix (DCM), also known as the orientation (attitude) matrix, along with its transpose, facilitates the transformation of vector representations between the earth-fixed frame and the body-fixed frame [56]. Euler angles—yaw, pitch, and roll—offer a straightforward method to describe rotations around three axes sequentially [15]. DCM, being a 3 × 3 orthogonal matrix with a unit determinant, belongs to the three-dimensional special orthogonal group SO(3) of rotation matrices, while the Euler angle formulation involve three consecutive rotations through body-referenced Euler angles [56]. The Euler angle representation provides the advantage of decoupling the yaw angle (y) from the other two angles (roll and pitch), facilitating a linear observation model. This decoupling helps to prevent coupling errors, where changes in one angle can inadvertently affect the measurement of the others, leading to inaccuracies during orientation correction [72]. However, they are susceptible to gimbal lock, where the loss of one degree of freedom occurs when two of the three rotation axes align, limiting their effectiveness in some situations. Quaternions, on the other hand, offer a more compact and robust representation of 3D rotations. They are defined using four parameters: one parameter specifies the angle of rotation, while the remaining three define the axis of rotation [70]. The quaternion representation is particularly advantageous for capturing the orientation of an IMU (Inertial Measurement Unit) because it avoids the gimbal lock issue associated with Euler angles [70]. Although rotation matrices maintain orthogonality, numerical integration errors can degrade it, requiring corrective methods [56]. Quaternions offer a more stable and computationally efficient approach, minimizing the drift and maintaining consistency in orientation representation during integration [56,70]. Numerous studies have employed quaternions to represent this orientation [9,21,27,70,73,74,83].

Estimating orientation often involves integrating three-axis angular velocity data from gyroscopes, which is effective for dynamic movements [15,22,70,85]. In a study by Mariani, Rochat, Büla and Aminian [25], inclination was determined by integrating pitch angular velocity using a 3D rotation matrix updated through quaternion-based integration between consecutive foot-flat instances. However, gyroscopes are susceptible to drift over time due to gyroscopic bias and the cumulative error of numerical integration [56]. Accelerometers can estimate inclination relative to gravity in static conditions but introduce noise in dynamic scenarios, affecting accuracy [22]. Magnetometers can determine orientation relative to the Earth’s magnetic field, but they are sensitive to external magnetic fields. Achieving accurate three-dimensional orientation estimates require purposefully exploiting the complementary properties of gyroscopes, accelerometers, and magnetic sensors [56]. The initial conditions for integration can be derived from an absolute orientation estimate using only accelerometer and magnetometer measurements when the system is stationary [89]. When the sensor is stationary, its measured accelerations are solely from Earth’s gravity, defining the global z-axis. Using these gravity measurements and an arbitrarily defined horizontal axis perpendicular to gravity, the initial quaternion is determined, which is updated whenever the foot makes contact with the ground, and during movement intervals, the quaternion is updated by gyroscopic angular rates integration [87].

Sensor fusion methods combine data from accelerometers, gyroscopes, and magnetometers (or a subset of these) to enhance orientation accuracy. For instance, Laidig, Jocham, Guggenberger, Adamer, Fischer and Seel [9] utilize gyroscopic measurements for continuous orientation updates, which are combined with a quaternion-derived accelerometer-based gravity correction. During stationary periods (ZVI), accelerometer data estimate sensor orientation using gravitational acceleration, while during movement, quaternion updates integrate gyroscope-measured angular rates for continuous orientation tracking [87].

The most commonly sensor fusion techniques are Kalman filter-based and complementary filter-based methods [51]. The Kalman filter, regarded highly for its ability to handle uncertainty, is an iterative algorithm designed to estimate a system’s state based on noisy measurements collected over time. Specifically in orientation estimation, the discrete Kalman filter integrates gyroscope data to continuously update orientation information, enhancing the accuracy and stability of orientation estimates [72]. By combining the accelerometer, gyroscope, and occasionally magnetometer readings, the Kalman filter aims to deliver precise and consistent estimates of orientation dynamics. Its operation necessitates a dynamic model of the system along with covariance matrices, enabling the prediction and adjustment of orientation throughout the observation period [49].

A smoother-based Kalman filter further refines orientation by integrating data from both forward and backward filters, thereby reducing orientation estimation errors by maintaining a smaller covariance throughout the process [72]. The Extended Kalman Filter (EKF) is specifically designed to handle nonlinear systems by employing linearization to approximate covariances and estimates errors in the acceleration, velocity, and position of human foot motion using an error measurement vector, helping mitigate IMU drift and noise, particularly in environments affected by local magnetic disturbances [86].

Complementary filters combine gyroscope and accelerometer data to estimate orientation. The complementary filter for attitude estimation from IMU readings applies high-pass filtering to gyroscope-based orientation estimates affected by low-frequency noise and low-pass filtering to accelerometer data affected by high-frequency noise, aiming to achieve an all-pass and noise-free attitude estimation through their fusion [90]. They are computationally less intensive than Kalman filters and offer good accuracy for many applications. Mahony and Madgwick are two popular complementary filter methods. The Madgwick filter is a gradient descent-based complementary filter designed to estimate orientation using data from both accelerometers and magnetometers, representing the orientation with quaternions [26,27,87]. Madgwick’s algorithm compensates for magnetic distortion without requiring predefined reference magnetic field directions. It is computationally less expensive than Kalman or extended Kalman filters and supports real-time data processing on a microcontroller [26]. The Mahony filter integrates accelerometer and gyroscope data while correcting for discrepancies between measured Earth-fixed vectors (gravity and magnetic field) and their estimated values based on current orientation. This correction, weighted by a parameter, enhances accuracy by incorporating magnetic readings into both attitude and heading estimation [89].

Kalman filter-based methods are renowned for their high accuracy in estimation tasks, but they are computationally demanding, while complementary filter-based methods are known to be computationally light and fairly accurate. Extended Kalman filters offer enhanced accuracy for nonlinear systems but require more processing time for each sample, taking about two orders of magnitude longer in embedded systems. Moreover, an extended Kalman filter approach necessitates a higher sampling rate, typically exceeding 250 Hz, to effectively capture and process rapid changes in dynamic systems, limiting its applicability in embedded systems where lower sampling rates are preferred for efficiency and resource conservation [91].

Particle filters, though less commonly used, enhance the estimation of foot trajectory by employing a cloud of particles to track the probability distribution of the state, dynamically updating and resampling possible states to provide reliable analysis of gait metrics [21]. Particle filters are advantageous when the error distribution of foot orientation estimation deviates significantly from a Gaussian distribution. Unlike traditional methods that assume Gaussian errors, particle filters excel in modeling and handling such non-Gaussian distributions, making them a preferred choice under these circumstances [21].

#### 6.1.4. Velocity and Position Estimation

Once the orientation is known, the accelerometer measurements can be transformed from the body frame to the navigation frame. This transformation is crucial for accurately interpreting the IMU’s readings relative to an external, fixed coordinate system. Gravity effects were removed by aligning accelerometer axes with the fixed frame and subtracting the gravity vector [15,22]. Specifically, the standard gravitational acceleration of 9.81 m/s^2^ is subtracted from the accelerometer readings. Aligning accelerations to a fixed frame and eliminating the gravitational effect along the vertical axis facilitated the extraction of the vertical trajectory through double integration in the fixed frame [25]. Without this adjustment, the inclusion of the gravitational acceleration (9.81 m/s^2^) in the process of acceleration integration would cause substantial errors to accumulate throughout the stride duration. The gravity-removed accelerations are then integrated once to obtain velocity and twice to obtain position [15,21,70,83,85].

#### 6.1.5. Dedrifting

Integrating acceleration data often results in significant integration drift, particularly with double integration. To mitigate this issue, researchers have introduced the zero-velocity update (ZUPT) technique, which assumes zero velocity and displacement during mid-stance periods. The approach separates individual strides by resetting the sensor’s position and orientation at the mid-stance. While this results in the loss of absolute position in space, it enhances the accuracy of computing individual stride parameters. This improvement stems from integrating data over shorter intervals, thereby reducing cumulative errors. An adaptation of this principle, known as the zero displacement update (ZDU), detects the flat foot phase and resets both vertical velocity and displacement to zero [15]. By detecting mid-stance or flat foot segments across multiple strides, this method aims to minimize the accumulation of drift errors between successive strides. However, at the end of each stride, a local drift occurs as an error between integrated and theoretical data zero, which is subsequently corrected [15].

The linear dedrifting technique estimates and subtracts a linear drift function from the estimated velocity, from which the displacement is obtained by integrating the corrected velocity [87]. The core idea behind dedrifting is based on the assumption that velocity remains zero during mid-stance. Using the first and last velocity values of the stride, a linear function was determined and applied to the velocity signal in all three directions [91]. Alternatively, some studies employ a p-chip interpolation function to model a sigmoid-like curve for drift removal [22,25].

The direct and reverse integration method integrates forward and backward in time, ensuring zero-velocity conditions at the interval boundaries. Results from both integrations are combined using a sigmoid weighting function to estimate accurate velocities and positions [87].

Suzuki, Hahn and Enomoto [70] employed a spatial error correction method to rectify errors and compared it with linear dedrifting based on velocity. The described method ensures that at the end of each gait cycle, the foot returns to a known reference orientation and position, allowing for the precise tracking of its trajectory and rotations. In the spatial error correction method, the yaw angle was calculated using the arctangent of the estimated mediolateral and anteroposterior positions of the foot. Similarly, the pitch angle was recalculated. Due to the nonlinear relationship between the pitch angle and vertical position, a gradient descent method was employed to find the pitch angle that nullifies the vertical position, enabling the accurate determination of foot orientation and position. The use of yaw and pitch angles ensures the foot’s orientation is correctly accounted for, while the gradient descent method ensures accurate recalibration of the pitch angle [70].

A smoother-based drift correction method utilizes both constant and piecewise-constant variance models to manage uncertainty in sensor measurements over time. The constant model maintains a steady variance, leading to linear increases in velocity covariance and linear corrections, whereas the piecewise-constant variance model also manages uncertainty but allows for varying levels across segments, leading to piecewise-linear increases in velocity covariance and corrections [72].

An error state Kalman filter with a Rauch–Tung–Striebel (RTS) smoother performs a backward pass on the estimated state to eliminate accumulated errors, iterating until it reaches the previous correction point, typically the previous zero-velocity phase [21].

Fukushi, Huang, Wang, Kajitani, Nihey and Nakahara [27] mitigated the drift components observed in the resultant velocity assuming linear motion with two rotations applied to preserve the trajectory’s natural shape.

#### 6.1.6. Machine Learning for Spatiotemporal Estimation

Machine learning techniques are used for designing algorithms that either learn from labeled data or detect a useful pattern in given data points and are being increasingly used in healthcare to support decision making. Recent research is increasingly shifting toward machine learning (ML) due to its high accuracy and ability to process gait parameters effectively, which is tailored to specific application requirements. Machine learning has been used in gait analysis to identify cerebral palsy [92], Parkinson’s disease [93], stroke [93], and functional gait disorders [94]. Machine learning approaches like decision trees [95], support vector machines [96], Bayesian networks [97], linear discriminant analysis [98], k nearest neighbors [99] and deep learning from raw IMU data [100,101] have been used for gait recognition applications. Recent advancements in machine learning techniques, including supervised learning, clustering-based unsupervised learning, and reinforcement learning, are increasingly applied to gait analysis [102]. While using IMUs, these algorithms analyze the movement data such as acceleration and angular velocity to extract patterns and insights related to movement dynamics, gait analysis, activity recognition, and health monitoring, benefiting biomechanics, sports performance analysis, rehabilitation, and personalized healthcare.

Machine learning (ML) enables strong and rapid classification by extracting simpler functions through high-time and nonlinear biomechanical knowledge. Unlike conventional threshold-based techniques, dynamic strategies are more complex, instinctive, flexible, and adaptable [103]. In contrast, walking evaluation through traditional methods is simplified, yet it remains subjective, lacking reliability, accuracy, and precision in assessing observer characteristics [103]. Machine learning has some drawbacks, including a lack of explainability, which makes it difficult to understand how models arrive at their decisions [103]. It also requires significant memory resources and computational power, especially for complex models. Additionally, ML relies on large, high-quality datasets for training, which can be challenging to obtain, particularly in specialized applications like gait analysis [104]. By overcoming data limitations and optimizing computational efficiency, ML could significantly improve the functionality and precision of these systems, making them more effective in gait analysis, injury prevention, and overall health monitoring.

Santhiranayagam, Lai, Sparrow and Begg [28] developed a Generalized Regression Neural Network (GRNN) machine learning model to estimate minimum toe clearance from IMUs. This method integrated features from both raw and integrated inertial signals to train Generalized Regression Neural Network (GRNN) models using a hill-climbing feature-selection method, demonstrating potential for real-time monitoring in everyday locomotion.

Guimarães, Sousa and Correia [74] developed a deep recurrent neural network to estimate heel and toe trajectories from which an extensive set of spatiotemporal gait parameters are estimated. Their approach utilized a dataset [87] with the acceleration and angular rate from two IMU sensors on shoe dorsum, which were synchronized with marker trajectories from an optical motion capture system. Preprocessing steps included segmenting continuous recordings, transforming coordinates, padding sequences to a fixed length, and normalizing data from both feet equally. A coordinate frame transformation for stride trajectories ensured independence from previous strides, which was supported by data augmentation through rotations for model robustness. Their stacked bidirectional LSTM (long short-term memory) recurrent network, consisting of two bidirectional LSTM layers, effectively handled domain-specific constraints, requiring inertial sensor data segmentation and normalization before making predictions.

### 6.2. Evaluation

Evaluation of gait analysis systems often relies on marker-based optical motion capture as a benchmark [10,15,22,25,26,27,70,72,83,85,86]. The Vicon motion capture system is used as the gold standard by the majority of the studies reviewed in this research [10,15,22,25,26,27,70,83]. As an alternative to motion capture systems, some research employed Microsoft Kinect [21] or instrumented treadmills [9] as the reference systems. In cases where validation against a gold standard is not conducted, evaluations may rely on intrinsic known values obtained during the activity itself [21,73]. This approach assesses the performance or effectiveness of a system or method based on observable outcomes rather than direct comparison to a highly accurate reference standard such as marker-based motion capture.

Most studies conducted evaluation using healthy adults as participants [10,15,22,25,26,27,70,83,85]. Additionally, while some researchers conducted tests at the user’s comfortable walking speeds [26], others tested at different walking speeds [15,83,86] and varying stride lengths [85] to assess algorithm effectiveness. Typically, markers are affixed to shoes to assess spatiotemporal characteristics alongside data from IMUs [10,27,70,87]. Researchers commonly use varying numbers of cameras for gait analysis, typically ranging from 7 to 16 [10,27,70,87]. Experiments involved participants walking either in a straight path [27] or on a treadmill [10]. Variations in experimental design included the length of the walking path, whether data were collected in one direction only [27] or in both directions [72]. Some researchers conducted experiments in both indoor and outdoor environments to test suitability across different settings [86].

Ensuring the synchronization of data between the two systems—IMU and the reference system—is crucial for accurate comparison and reliable evaluation. To synchronize the IMU and Vicon systems for comparing gait parameters, Guimarães, Sousa and Correia [87] employed cross-correlation between the acceleration magnitude from the IMU and the centroid trajectory of sensor markers from Vicon. The centroid trajectory was derived twice to ensure accuracy, and the maximum cross-correlation value was used to compensate for any time-shift between the data sources [87]. Another synchronization method uses timestamping, where Bluetooth Low Energy (BLE) transmits data wirelessly from the IMU to a smartphone. Each data packet is tagged with a timestamp indicating when it was received, allowing accurate correlation with timestamped signals from the motion-capture system [27]. A different technique used a wireless trigger system, which employs a transmitter at a light barrier and a receiver at the IMU input, activating an impulse upon the subject crossing the barrier while maximizing cross-correlation between the foot angle, heel, and toe clearance signals across both measurement systems for each stride [83].

The evaluation methods for IMU-based gait analysis systems vary depending on the specific needs and constraints of each study. Researchers must carefully choose their evaluation approach based on factors such as the desired accuracy, time, and cost limitations, as well as the specific applications of the system. While many studies utilize marker-based optical motion capture systems like Vicon as the gold standard, alternatives such as Kinect and instrumented treadmills are also valuable in specific contexts. The evaluation of IMU-based systems for gait analysis highlights the importance of synchronization with high-precision reference systems, such as Vicon, to ensure accurate data alignment and comparison. Although most studies focus on healthy adults, there is a need to expand validation efforts to include more diverse populations and real-world settings, which would further establish the reliability and utility of these systems for clinical use and everyday applications.

## 7. Conclusions

The desire to enhance quality of life, facilitate reliable rehabilitation, and gather valuable kinetic and kinematic insights across various applications motivates ongoing research in gait analysis. The increasing need for lightweight, portable, and wireless tools for gait assessment in natural environments highlights the role of wearable sensors as an alternative to laboratory-based systems. Accurate three-dimensional foot trajectory estimation is essential across numerous applications, often requiring precise IMU orientation estimation to minimize integration drift in accelerometers and gyroscopes. Despite zero-velocity update (ZUPT) corrections helping stabilize orientation and velocity drifts, integration drifts remain a challenge, affecting position estimates.

This review explored various methods to mitigate these errors, emphasizing recent advancements that have enhanced the accuracy of IMU-based foot trajectory estimation. Calibration-free and orientation-free approaches show promise for reliable gait analysis with minimal setup, increasing usability in clinical and everyday environments. These innovations are critical for developing wearable technologies aimed at fall prevention and gait monitoring, providing real-time insights into gait dynamics for more effective risk assessments.

Research into algorithms for detecting gait parameters using IMU sensors has evolved, exploring diverse methods to improve accuracy and usability. Future studies could explore the application of machine learning models for real-time fall risk prediction based on gait analysis. Building on recent advancements in calibration-free methods, further research could aim to develop and validate machine learning algorithms that require minimal or no calibration. Simplifying the deployment of gait analysis systems could make them more accessible and practical in diverse settings. Evaluating algorithms under varied conditions—such as different walking speeds, environments, and stride lengths—is essential to enhance their robustness. Ensuring synchronization between motion capture systems and newly developed ones is vital for maintaining the accuracy and reliability of evaluations, allowing for consistent and meaningful comparisons across different systems and advancements.

In conclusion, future work should focus on refining these methods and expanding their applications, ultimately enhancing the effectiveness of wearable technologies for monitoring gait and preventing falls in a range of real-world settings.

## Figures and Tables

**Table 1:** Illustration of gait shoes using wearable sensors.

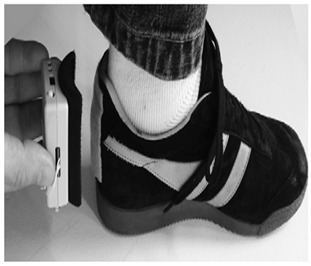 S—sense module attached to shoe [22]	Sensor: Six-axis inertial measurement unit Sensor Location: Hind-foot position Functionality: Detects temporal parameters, combined with optimized fusion and de-drifted integration of inertial signals. The system computes stride length, stride velocity, foot clearance, and turning angle parameters for each gait cycle, derived from the analysis of 3D foot kinematics.
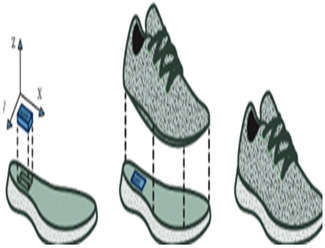 Nushu System [26]	Sensor: Inertial Measurement Unit (IMU) Sensor Location: Posterior portion of the outsoles Functionality: Real-time gait monitoring with in-shoe system. Investigated the optimal sampling frequency to capture all the information on walking pattern.
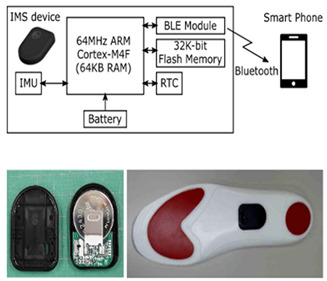 In-Shoe Motion Sensor System [27]	Sensor: Inertial Measurement Unit (IMU) Sensor Location: Insole of shoe Processor: Cortex-M4 ultralow-power advanced RISC machine (ARM) processor Functionality: On-line stride segmentation based on stable foot-flat detection. Three-dimensional zero-velocity update for accurate stride parameterization.
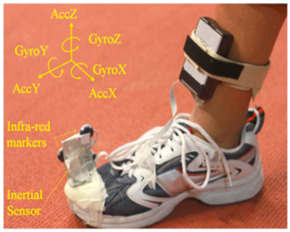 Minimum Toe Clearance Estimation [28]	Sensor: Inertial Measurement Unit (IMU) Sensor Location: Distal foot Functionality: Developed a machine learning approach for real-time estimation of minimum toe clearance height during everyday locomotion.
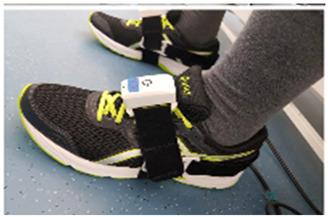 Calibration-Free Gait Assessment [9]	Sensor: Inertial Measurement Unit (IMU) Sensor Location: Dorsum position of each shoe. Functionality: Does not require calibration or precise mounting of sensors. Determine spatiotemporal gait parameters essential for clinical gait assessment. Unsupervised use by non-experts in indoor as well as outdoor environments.

**Table 2:** Examples of healthy spatiotemporal gait parameter ranges across different age brackets. Adapted from [36].

Parameters (Self-Selected Pace)	Young (1–7)	Adult	Older Adults (>65)
Walking velocity (m/s)	0.64–1.14	1.30–1.46	Declines 15% per decade
Stride length (m)	0.23–0.57	1.68–1.72	1.66–1.70
Step length (m)	0.20–0.32	0.68–0.85	0.44–0.60
Stance phase (s)	0.32–0.54	0.62–0.70	0.68–0.72
Swing phase (s)	0.19–0.27	0.36–0.40	0.42–0.44
Cadence-fast walking (steps/min)	176–144	113–118	58–70
Single support (% of stride)	64.4–65.6	60.6–62.0	61.7–62.9
Double support (% of stride)	22.5–23.9	21.2–23.8	23.4–25.8

**Table 3:** Comparison of different wearable sensors used for gait analysis [51,53].

Sensor Type	Parameter Measured	Location Attached	Advantages	Disadvantages
Foot Switch	Timing of gait events such as heel strike and toe-off	Heel or sole	Low-cost, simple signal conditioning and postprocessing Provides precise timing information for gait events	Limited number of the detectable gait phases, accuracy and reliability depends on the placement of the sensors. Wire connections can decrease the system service life. Force generated in the gait cycle cannot be isolated by the concurrent effects induced by the movement of the center of mass.
Insole Pressure Sensor	Pressure distribution of foot	Embedded in insole of shoe	Provides detailed information on foot pressure distribution during walking Can help in assessing foot function and detecting abnormalities in gait. Relatively simple to use and interpret.	May not capture dynamic changes in pressure distribution during walking. Subject to pressure between sensor and the foot, leading to non-zero pressure readings even in swing phase. Short life span.
Linear Accelerometer	Acceleration	Shank or thigh for limb movement, Trunk for whole body movement	Measure acceleration, providing information on movement patterns and intensity. Portable and wearable, allowing for natural movement during analysis.	Require calibration and careful placement for accurate measurements. Subject to influence of gravity and sensitive to position and orientation. Limited to measuring acceleration along specific axes, may not capture full-body motion.
Gyroscope	Angular Velocity	Shank or thigh	Measure angular velocity, providing information on rotational movement. Less prone to noise and invariant to translation in position. Can be used in combination with accelerometers for more comprehensive motion analysis.	Require calibration and careful placement for accurate measurements. May be sensitive to drift over time, requiring periodic recalibration.
Inertial Measurement Units	Acceleration and angular velocity, magnetic field strength	Foot, shank, knee, thigh, pelvis, head, upper limb or other parts for motion analysis	Portable, low energy consumption, low cost, durable, and reliable. Can provide real-time data on acceleration, angular velocity, and orientation.	May require complex calibration procedures for accurate measurements. Limited to measuring motion at the sensor location, which may not capture whole-body movement. Magnetometer is sensitive to external magnetic field. Prone to drift over time.
EMG	Muscle activation levels, timing and duration of muscle contractions	Specific muscles of interest	Measure muscle activity during walking, providing insights into muscle function and coordination. Can help in assessing muscle imbalance and gait abnormalities.	Requires skin preparation and placement of electrodes on the skin, which can be uncomfortable and may limit natural movement. Complex data acquisition and processing steps. Sensitive to moisture between skin and sensor.

**Table 4:** Features of smart shoe systems utilizing IMUs for foot trajectory and spatiotemporal estimations. RMSE: root mean square error.

System Name	Number of IMUs	Sensor Location	Algorithm					Estimated Parameters	Results	Validation	
			Preprocessing	Gait Segmentation	Orientation Estimation	Displacement Estimation	Drift Correction			Reference System	Participants
**Measuring Highly Accurate Foot Position And Angle Trajectories, 2024** [10]	2: One on each shoe	Instep/Dorsum of each shoe	*Not used*	Threshold	Quaternion-based gyroscope strapdown integration	Double integration of acceleration	Linear dedrifting	Stance duration, swing duration, stride length, walking speed, and cadence	Average RMSE of 0.67° for pitch, 0.63° for roll and 1.17° for yaw. For position trajectories, 0.51 cm for vertical lift and 0.34 cm for lateral shift	Vicon with 16 cameras, participants walking on treadmill	23 healthy adults
**Running Trajectory Estimation, 2022** [70]	2: One on each foot	Dorsum of foot	Quintic spline function low-pass filter	Peak detection on resultant acceleration	Quaternion-based gyroscopic strapdown integration	Double integration of acceleration	Spatial error correction (SEC)/linear dedrifting (LD)	Foot trajectory, Stride length	SEC method estimated stride length more accurately compared to the LD method	16 camera Vicon MX system	79 runners
**In-Shoe Motion Sensor System, 2022** [27]	1	Insole	Sampling at 100 Hz	Foot-sole angle threshold technique with temporal window	Madgwick filter	Double integration of acceleration	Two rotations	Stride length, walking speed, foot height, circumduction, peak foot Sole angle in the dorsiflexion and plantarflexion directions and toe-in/out angle	RMSE for stride length: 0.069 m, walking speed: 0.094 m/s, foot height: 1.5 cm, circumduction: 1.0 cm, peak foot-sole angle in dorsiflexion: 3.3 deg. and in the plantarflexion directions: 5.9 deg., toe-in/out angle: 2.5 deg	Track 3 (Vicon Motion Systems) with 10 cameras	30 healthy adults
**Nushu System, 2021** [26]	1	Posterior portion of the outsoles	Anti-aliasing filter	Dynamic thresholds and local peak-identification on angular velocity	Madgwick filter	Double integration of acceleration	ZUPT and linear dedrifting	Stride velocity, stride time, stride length, minimum foot clearance, strike angle, stance time, swing time, stance phase, swing phase, cadence, maximum angular velocity, symmetry, and variability	Percentage mean absolute errors (MAE%) stride time: 1.19%, stride length: 1.68%, stride velocity: 2.08%, cadence: 1.23%,	Vicon motion capture system with 14 cameras	4 healthy adults
**Calibration Free Gait shoes, 2021** [9]	One IMU on each foot	Dorsum of shoe	*Not mentioned*	Automatic adaptive threshold	Quaternion-based gyroscope strapdown integration and moving average filer on acceleration	Double integration of acceleration	ZUPT and linear dedrifting	Swing duration, stance duration, stride length, walking speed, cadence	Mean absolute difference is 1.4% for the gait phase durations, 1.7 cm for the stride length, 0.04 km/h for the walking speed, and 0.7 steps/min for the cadence	Zebris Rehawalk instrumented treadmills (Zebris Medical, Isny, Germany)	137 (39 healthy, 36 with different neurological diseases, 62 orthopedic diseases)
**Smoother-Based 3D system, 2019** [72]	One IMU on each foot	Heel of Shoe	*Not mentioned*	Threshold method	Smoother-based Kalman filter with Euler angle representation	Double integration	Smoother-based correction	Foot trajectory	Accuracy on strid length: −0.24 ± 1.11 cm and on stride width: −0.02 ± 0.95 cm. RMS errors reduction by 62% on stride length and 44% on stride width	Motion analysis, 8 cameras	9 adults
**Inertial Sensor Based Robust Gait Analysis, 2017** [21]	One IMU on each foot	Dorsum of foot	Sampling rate of 100 Hz	Threshold on angular velocity	Particle filter	Double integration of acceleration	Error state Kalman filter with Rauch–Tung–Striebel smoother	Stride length, cadence, cycle time, stance time, swing time, stance ratio, speed, maximum/minimum clearance and turning rate	Capture various neurological disorder-related gait abnormalities in a non-hospital setting	Microsoft Kinect depth-camera and a slow-motion camera	1st phase: 22 (16 healthy) subjects, 2nd phase: 17 subjects with neurological disorder
Real-Time Foot Motion with DYNAMIC SPEED, 2016 [86]	1	Dorsum of foot	*Not mentioned*	Dynamic gait phase detection	Extended Kalman filter	Double integration of acceleration	Extended Kalman filter, heuristics heading reduction, zero-velocity update	Foot position, velocity and attitude	Accurately estimate, in real time, the human foot position, velocity, and attitude in dynamic motion speeds. High-accuracy localization both indoors (0.375% errors) and outdoors (0.55% errors)	Motion Analysis Corporation with 16 cameras	*Not mentioned*
**3D Tracking Shoe Sensing, 2016** [73]	1	Upper part of shoe	*Not mentioned*	Threshold on acceleration energy	Quaternion with fourth-order Runge–Kutta method	Double integration of acceleration	Linear error dedrifting	3D tracking and vertical height estimation	Error Walking: 0.40% Jogging: 0.36% Upstairs: 0.56% Downstairs: 0.88%	*Not mentioned*	*Not mentioned*
**Estimation of Foot Trajectory During Human Walking, 2016** [85]	1	Dorsum of foot	Low-pass filter, sampled at 200 Hz	Threshold on angular velocity for a specific time period	Integration of angular velocity	Double integration	Zero displacement correction	Stride length, foot clearance	Mean accuracy and precision 20 ± 50 mm, for stride length, and 2 ± 7 mm for foot clearance	MAC3D System: Motion Analysis Corporation, USA) with 8 cameras	10 healthy adults
**Robust Foot Clearance Estimation, 2016** [15]	2: One on each foot	Ankle joint	Calibration, first-order Butterworth filter	Minimum of angular velocity	3D gyroscopic integration	Double integration of acceleration	Zero displacement update	Foot clearance	Accurate foot clearance estimation, with normalized root mean square errors (NRMSE) below 15% in 96% of cases, and root mean square (RMS) errors generally below 1.5 cm across various walking conditions	Vicon motion capture	10 healthy adults
**Inertial Sensor Based and Shoe Size Independent, 2015** [83]	2: One IMU on each foot	Lateral ankle	*Not mentioned*	Subsequence dynamic time wrapping on angular velocity and acceleration	Gyroscopic integration, quaternion representation	Double integration of acceleration	Piecewise cubic Hermite interpolating polynomial	Continuous heel and toe clearance	High correlation and low mean absolute error for both toe clearance (1.69 ± 0.70 cm) and foot angle (2.49 ± 1.21°)	Vicon motion capture with 16 cameras	20 healthy participants
**Orientation Invariant Gait Analysis, 2021** [87]	2: One IMU on each foot	Foot instep	Zero-lag bidirectional 2nd order Butterworth low-pass filter	Zero velocity interval and peak detection	Quaternion representation, tested gyroscope integration, Madgwick filter and Euston filter	Double integration of acceleration	Linear dedrifting, direct and reverse integration	Swing and stance duration, cadence, stride length, stride width, minimum toe clearance	High accuracy, with correlations ranging from 0.78 to 0.98 and relative errors between 1.4% and 7.9% for key gait metrics	Vicon motion capture with 10 cameras	26 healthy adults
**Physilog Unit, 2012** [25]	1	Upper part of shoes	Low-pass filter	Peak detection	2D: Integration of pitch 3D: quaternion-based time Integration of the angular velocity	Double integration of acceleration	P-chip linear interpolation	Foot clearance	Accuracy ± precision of 4.1 ± 2.3 cm for maximal heel clearance and 1.3 ± 0.9 cm for minimal toe clearance	Vicon motion capture system with 7 cameras	12 healthy adults
**S-Sense, 2010** [22]	2: One on each shoe	Hind foot	Low-pass filter at 17 Hz, sampled on 12 bits at 200 Hz	Threshold detection on angular velocity	Acceleration-based inclination and azimuth determination	Double integration of acceleration	P-chip linear interpolation	Stride length, stride velocity, foot clearance, and turning angle parameters	Mean accuracy ± precision was 1.5 ± 6.8 cm for stride length, 1.4 ± 5.6 cm/s for stride velocity, 1.9 ± 2.0 cm for foot clearance, and 1.6 ± 6.11 for turning angle	Vicon motion capture	20 healthy adults

**Table 5:** Significance and limitations of the reviewed smart shoe systems.

Study Name	Significance	Limitations
Measuring Highly Accurate Foot Position and Angle Trajectories [10]	Accurate measurements of foot position and angle trajectories with low errors. Easy to use without requiring precise mounting, calibration, or magnetometer data. Consistent accuracy for both normal and pathological gait. Provides an easy-to-use alternative to optical motion capture, facilitating gait analysis outside laboratory settings. Investigates the influence of shoe motion as a secondary outcome.	Findings are based on healthy individuals, which may not fully represent gait pathologies. Treadmill walking might not fully represent natural gait, affecting generalizability. Simulated gait pathology introduced differences in movement patterns between the left and right sides. Slightly larger MAD for the right yaw angle due to potential calibration inaccuracies in the IMU.
Running Trajectory Estimation [70]	Consistent performance across a range of running speeds, making it a reliable tool for level ground running analysis. SEC method provides a more accurate estimation of stride length and foot trajectory, effectively addressing IMU drift.	SEC method is limited to level ground running and might not be applicable to faster speeds or different environments. Results should be validated in outdoor settings.
In-Shoe Motion Sensor System [27]	Proposed on-line algorithms for stride parameter estimation. Provides immediate feedback and analysis during walking, crucial for real-time monitoring. Utilizes high-g detection for accurate walking bout identification and efficient power management. Minimizes battery consumption, extending usability and practicality for daily wear.	Stride-segmentation algorithm relies on typical foot-sole angle waveforms of healthy participants, limiting accuracy for the elderly or hemiplegia, osteoarthritis, or Parkinson’s conditions. System assumes straight and level walking, which may not perform well in varied environments such as speed changes direction shifts, stairs/slope ascent/descent, running, or obstacle navigation. Only detects foot contact and toe-off.
Nushu System [26]	Portable in-shoe gait analysis system with real-time Bluetooth transfer or local SD card storage. Wireless charging via an electromagnetic induction docking station. New intuitive visualization method using hodographs for describing spatiotemporal relations. Identified optimal sampling frequency to prevent aliasing and ensure accurate high-frequency gait signal capture. Achieved high accuracy in stride length and velocity compared to other wearable systems.	Validated only with limited small-scale healthy subjects; future plans include larger and more diverse populations. No free-living movements assessed; performance under varying conditions and speeds needs further validation. No synchronization between left and right sensors; future work could include synchronized recording.
Calibration Free Gait shoes [9]	Proposed algorithms do not require magnetometers, precise sensor mounting, or dedicated calibration movements. Suitable for unsupervised use by non-experts in indoor and outdoor environments.	No stride-by-stride comparison due to reference system limitations. Recordings were made on treadmills, which may differ from overground walking.
Smoother-Based 3D system [72]	Stride length and width errors reduced by 62% and 44%, respectively. Both orientation and velocity estimations are enhanced, leading to better trajectory accuracy. Fusing forward and backward Kalman filters improves accuracy. Using a piecewise linear function refines velocity estimates and reduces drift errors.	Focused only on the stride length and stride width of ground walking. Velocity estimation needs adjustment for different gaits and drifting models.
Inertial Sensor-Based Robust Gait Analysis [21]	The system detects side, back, and turning steps, beyond straight walking. Introduces a new lateral stride profile visualization method for instant spatial gait insights. Effectively captures gait abnormalities related to neurological disorders in non-hospital settings. Tested with patients with neurological disorders.	Lacks comparison with a gold standard reference system. Initial studies focused on a small dataset and specific conditions.
Real-Time Foot Motion with Dynamic Speed [86]	Provides accurate real-time dynamic gait phase detection of human foot motion, addressing both heading drifts and magnetic noise. Effective across different walking speeds and environments, including indoor and outdoor settings.	Performance with embedded wearable processors has not yet been evaluated.
3D Tracking Shoe Sensing [73]	Provides accurate real-time indoor 3D positioning and tracking of users’ movements, including walking, jogging, and navigating stairs. Addresses cumulative sensor errors by leveraging the linear property of acceleration error drift. Includes a walking state classification model and real-time 3D trajectory mapping, expanding its application to various movement scenarios. Designed for easy integration into wearable technology, specifically shoes, enhancing its practical use in everyday settings.	Lacks comparison with a gold standard reference system.
Estimation of Foot Trajectory During Human Walking [85]	Real-time 3D gait monitoring system beneficial for fall risk prediction and prevention in older adults. Adaptable to different walking surfaces; can detect stairs and flat floors using sensor signals.	Assumes a flat walking surface, not accurate on slopes or uneven terrain. Tested only on young adults; needs further evaluation with elderly subjects for fall prevention applications of elderly.
Robust Foot Clearance Estimation [15]	Designed to be robust against misalignment between the IMU and the foot axes. Good accuracy in estimating foot clearance across different walking conditions (normal, fast, and walking with obstacles). Although the current method is performed offline, it has potential for adaptation to online, real-time applications. Validated against motion capture system with different subjects having varying sizes and gaits, demonstrating its generalizability and potential use across diverse populations.	Performance degradation at higher walking speeds, likely due to the IMU’s acquisition frequency constraints when handling high accelerations. Reduced accuracy in estimating foot clearance during less predictable, non-periodic walking types. Method was validated offline with data acquired on an SD card, requiring adaptation for real-time, wireless applications. Sensitive to sensor noise and alignment in double integration and drift correction.
Inertial Sensor-Based and Shoe Size Independent [83]	Shoe size-independent approach for continuous heel/toe clearance estimation. High correlation (0.98) and low mean absolute error (1.69 ± 0.70 cm) for toe clearance. Validated with real gait data, showing high correlation (0.99) for foot angle estimation. Age-independent performance for all gait parameters. Mobile, unobtrusive system suitable for home monitoring or clinical use. Potential to assess fall risk through discrete features from continuous clearance signals.	Higher errors for higher clearance values due to rigid shoe model not accounting for natural bending during walking. Parameterized kernels for smoothing limit generalization and adaptation to new datasets. Lack of cross-validation reduces robustness for new data. Method not evaluated for different shoe types, limiting broader applicability.
Orientation Invariant Gait Analysis [87]	Reliable gait analysis without requiring precise sensor alignment on the foot. Horizontal correction mechanisms used in the integration process improve accuracy when walking on flat surfaces. Provides accurate gait metrics with a low error rate (1.4–7.9%).	Assumption of flat walking surface for horizontal corrections and cannot generalize to inclined surfaces or stair climbing. Higher relative errors in estimating MTC. Errors in stride length and speed increase when turns are included.
Physilog Unit [25]	First study to use wireless inertial sensors validated against a reference system for estimating foot clearance parameters. Provides accurate and precise estimation of foot clearance parameters, outperforming previous inertial-based systems. Enables gait analysis outside the lab in natural conditions, providing a lightweight, easy-to-use system. Potential applications in clinical settings for assessing mobility in elderly populations or individuals with neurological conditions.	Assumes normal gait patterns with heel-strike and toe-off; may not work for specific abnormal gait patterns. Potential errors due to sensor calibration, drift from double integration, and manual placement of markers. Sensor location estimation may introduce variability, and the foot model’s simplifications do not account for deformations during gait. Requires further validation in clinical settings and with different protocols, especially during turns or complex gait scenarios.
S-sense [22]	Wearable system with a 3D gait assessment algorithm, validated against an optical motion capture system. Demonstrates high test-retest reliability and adaptability to different foot positions, enhancing usability. Provides new gait parameters like turning angle (TA) and foot clearance (FC), aiding fall risk assessment in clinical settings. Lightweight, non-intrusive design enables natural gait analysis, suitable for daily use in research and rehabilitation. Offers comparable or better accuracy for stride length (SL) and stride velocity (SV) compared to other inertial-based systems, supporting its utility for clinical evaluations.	Wireless functionality suffers from a 2% frame loss; consecutive frame loss could significantly impact accuracy, suggesting a need for on-device recording. Sensor saturation with accelerations above 3g can cause errors, especially in younger, more active users. Azimuth (heading) drift can affect long-term trajectory, requiring additional sensors (magnetometer, GPS) for improved orientation, though these come with their own limitations. Further research is needed to validate the system in frail elderly populations and to assess the impact of age, gender, and variability estimations on gait analysis. Accuracy may be affected by centrifugal acceleration during rotation and environmental factors affecting additional sensors (e.g., magnetometers).

## Data Availability

Data are contained within the article.

## References

[1] Hegde N., Bries M., Sazonov E. (2016). A Comparative Review of Footwear-Based Wearable Systems. Electronics.

[2] Argañarás J.G., Wong Y.T., Begg R., Karmakar N.C. (2021). State-of-the-art wearable sensors and possibilities for radar in fall prevention. Sensors.

[3] Carse B., Meadows B., Bowers R., Rowe P. (2013). Affordable clinical gait analysis: An assessment of the marker tracking accuracy of a new low-cost optical 3D motion analysis system. Physiotherapy.

[4] Henmi S., Yonenobu K., Masatomi T., Oda K. (2006). A biomechanical study of activities of daily living using neck and upper limbs with an optical three-dimensional motion analysis system. Mod. Rheumatol..

[5] Karaulova I.A., Hall P.M., Marshall A.D. (2002). Tracking people in three dimensions using a hierarchical model of dynamics. Image Vis. Comput..

[6] Guimarães V., Sousa I., Correia M.V. Gait events detection from heel and toe trajectories: Comparison of methods using multiple datasets. Proceedings of the 2021 IEEE International Symposium on Medical Measurements and Applications (MeMeA).

[7] Mason R., Pearson L.T., Barry G., Young F., Lennon O., Godfrey A., Stuart S. (2023). Wearables for Running Gait Analysis: A Systematic Review. Sports Med..

[8] Tao W., Liu T., Zheng R., Feng H. (2012). Gait Analysis Using Wearable Sensors. Sensors.

[9] Laidig D., Jocham A.J., Guggenberger B., Adamer K., Fischer M., Seel T. (2021). Calibration-free gait assessment by foot-worn inertial sensors. Front. Digit. Health.

[10] Jocham A.J., Laidig D., Guggenberger B., Seel T. (2024). Measuring highly accurate foot position and angle trajectories with foot-mounted IMUs in clinical practice. Gait Posture.

[11] Wei W., Kurita K., Kuang J., Gao A. Real-Time Limb Motion Tracking with a Single IMU Sensor for Physical Therapy Exercises. Proceedings of the 2021 43rd Annual International Conference of the IEEE Engineering in Medicine & Biology Society (EMBC).

[12] Suh Y.S. (2014). Inertial sensor-based smoother for gait analysis. Sensors.

[13] López-Nava I.H., Muñoz-Meléndez A. (2016). Wearable Inertial Sensors for Human Motion Analysis: A Review. IEEE Sens. J..

[14] Beravs T., Reberšek P., Novak D., Podobnik J., Munih M. Development and validation of a wearable inertial measurement system for use with lower limb exoskeletons. Proceedings of the 2011 11th IEEE-RAS International Conference on Humanoid Robots.

[15] Benoussaad M., Sijobert B., Mombaur K., Azevedo Coste C. (2016). Robust Foot Clearance Estimation Based on the Integration of Foot-Mounted IMU Acceleration Data. Sensors.

[16] Pacini Panebianco G., Bisi M.C., Stagni R., Fantozzi S. (2018). Analysis of the performance of 17 algorithms from a systematic review: Influence of sensor position, analysed variable and computational approach in gait timing estimation from IMU measurements. Gait Posture.

[17] Patterson M.R., Johnston W., Mahony N.O., Mahony S.O., Nolan E., Caulfield B. Validation of temporal gait metrics from three IMU locations to the gold standard force plate. Proceedings of the 2016 38th Annual International Conference of the IEEE Engineering in Medicine and Biology Society (EMBC).

[18] Lindemann U. (2020). Spatiotemporal gait analysis of older persons in clinical practice and research. Z. Für Gerontol. Geriatr..

[19] Hamacher D., Hamacher D., Schega L. (2014). Towards the importance of minimum toe clearance in level ground walking in a healthy elderly population. Gait Posture.

[20] Begg R., Best R., Dell’Oro L.A., Taylor S. (2007). Minimum foot clearance during walking: Strategies for the minimisation of trip-related falls. Gait Posture.

[21] Tunca C., Pehlivan N., Ak N., Arnrich B., Salur G., Ersoy C. (2017). Inertial Sensor-Based Robust Gait Analysis in Non-Hospital Settings for Neurological Disorders. Sensors.

[22] Mariani B., Hoskovec C., Rochat S., Büla C., Penders J., Aminian K. (2010). 3D gait assessment in young and elderly subjects using foot-worn inertial sensors. J. Biomech..

[23] Tao S., Zhang X., Cai H., Lv Z., Hu C., Xie H. (2018). Gait based biometric personal authentication by using MEMS inertial sensors. J. Ambient. Intell. Humaniz. Comput..

[24] Jacob S., Fernie G., Fekr A.R. (2021). Design of a Novel Wearable System for Foot Clearance Estimation. Sensors.

[25] Mariani B., Rochat S., Büla C.J., Aminian K. (2012). Heel and Toe Clearance Estimation for Gait Analysis Using Wireless Inertial Sensors. IEEE Trans. Biomed. Eng..

[26] Wu J., Kuruvithadam K., Schaer A., Stoneham R., Chatzipirpiridis G., Easthope C.A., Barry G., Martin J., Pané S., Nelson B.J. (2021). An intelligent in-shoe system for gait monitoring and analysis with optimized sampling and real-time visualization capabilities. Sensors.

[27] Fukushi K., Huang C., Wang Z., Kajitani H., Nihey F., Nakahara K. (2022). On-Line Algorithms of Stride-Parameter Estimation for in-Shoe Motion-Sensor System. IEEE Sens. J..

[28] Santhiranayagam B.K., Lai D.T.H., Sparrow W.A., Begg R.K. (2015). A machine learning approach to estimate Minimum Toe Clearance using Inertial Measurement Units. J. Biomech..

[29] Perry J., Burnfield J.M. (2010). Gait analysis: Normal and pathological function. J. Sports Sci. Med..

[30] Kharb A., Saini V., Jain Y., Dhiman S. (2011). A review of gait cycle and its parameters. IJCEM Int. J. Comput. Eng. Manag..

[31] Vu H.T.T., Dong D., Cao H.-L., Verstraten T., Lefeber D., Vanderborght B., Geeroms J. (2020). A Review of Gait Phase Detection Algorithms for Lower Limb Prostheses. Sensors.

[32] Di Gregorio R., Vocenas L. (2021). Identification of Gait-Cycle Phases for Prosthesis Control. Biomimetics.

[33] Mustapha B., Zayegh A., Begg R.K. Ultrasonic and infrared sensors performance in a wireless obstacle detection system. Proceedings of the 2013 1st International Conference on Artificial Intelligence, Modelling and Simulation.

[34] Hausdorff J.M., Rios D.A., Edelberg H.K. (2001). Gait variability and fall risk in community-living older adults: A 1-year prospective study. Arch. Phys. Med. Rehabil..

[35] Maki B.E. (1997). Gait changes in older adults: Predictors of falls or indicators of fear?. J. Am. Geriatr. Soc..

[36] Srivises W., Nilkhamhang I., Tungpimolrut K. Design of a smart shoe for reliable gait analysis using state transition theory. Proceedings of the 2012 9th International Conference on Electrical Engineering/Electronics, Computer, Telecommunications and Information Technology.

[37] Best R., Begg R. (2008). A method for calculating the probability of tripping while walking. J. Biomech..

[38] Lamine H., Bennour S., Laribi M., Romdhane L., Zaghloul S. (2017). Evaluation of calibrated kinect gait kinematics using a vicon motion capture system. Comput. Methods Biomech. Biomed. Eng..

[39] Lai D.T., Taylor S.B., Begg R.K. (2012). Prediction of foot clearance parameters as a precursor to forecasting the risk of tripping and falling. Hum. Mov. Sci..

[40] Baskwill A.J., Belli P., Kelleher L. (2017). Evaluation of a gait assessment module using 3D motion capture technology. Int. J. Ther. Massage Bodyw..

[41] Dadashi F., Mariani B., Rochat S., Büla C.J., Santos-Eggimann B., Aminian K. (2014). Gait and Foot Clearance Parameters Obtained Using Shoe-Worn Inertial Sensors in a Large-Population Sample of Older Adults. Sensors.

[42] Mills P., Barrett R. (2001). Swing phase mechanics of healthy young and elderly men. Hum. Mov. Sci..

[43] Singh Y., Kher M., Vashista V. Intention Detection and Gait Recognition (IDGR) System for Gait Assessment: A Pilot Study. Proceedings of the 2019 28th IEEE International Conference on Robot and Human Interactive Communication (RO-MAN).

[44] Park S., Park F.C., Choi J., Kim H. EEG-based Gait State and Gait Intention Recognition Using Spatio-Spectral Convolutional Neural Network. Proceedings of the 2019 7th International Winter Conference on Brain-Computer Interface (BCI).

[45] Begg R., Lai D., Taylor S., Palaniswami M. SVM Models in the Diagnosis of Balance Impairments. Proceedings of the 2005 3rd International Conference on Intelligent Sensing and Information Processing.

[46] Khandoker A., Lai D., Begg R., Palaniswami M. (2008). Wavelet-Based Feature Extraction for Support Vector Machines for Screening Balance Impairments in the Elderly. IEEE Trans. Neural Syst. Rehabil. Eng..

[47] Lai D., Begg R.K., Taylor S., Palaniswami M. (2008). Detection of tripping gait patterns in the elderly using autoregressive features and support vector machines. J. Biomech..

[48] Caldas R., Mundt M., Potthast W., de Lima Neto F.B., Markert B. (2017). A systematic review of gait analysis methods based on inertial sensors and adaptive algorithms. Gait Posture.

[49] Wang Z., Yang Z., Dong T. (2017). A Review of Wearable Technologies for Elderly Care that Can Accurately Track Indoor Position, Recognize Physical Activities and Monitor Vital Signs in Real Time. Sensors.

[50] Zhou H., Hu H. (2008). Human motion tracking for rehabilitation—A survey. Biomed. Signal Process. Control..

[51] Prasanth H., Caban M., Keller U., Courtine G., Ijspeert A., Vallery H., von Zitzewitz J. (2021). Wearable Sensor-Based Real-Time Gait Detection: A Systematic Review. Sensors.

[52] Ishida T., Samukawa M. (2023). Validity and Reliability of a Wearable Goniometer Sensor Controlled by a Mobile Application for Measuring Knee Flexion/Extension Angle during the Gait Cycle. Sensors.

[53] Taborri J., Palermo E., Rossi S., Cappa P. (2016). Gait Partitioning Methods: A Systematic Review. Sensors.

[54] Bamberg S.J.M., Benbasat A.Y., Scarborough D.M., Krebs D.E., Paradiso J.A. (2008). Gait Analysis Using a Shoe-Integrated Wireless Sensor System. IEEE Trans. Inf. Technol. Biomed..

[55] Zhang Y., Wu Y., Nie J., Yu Y. Estimation of Continous Heel and Toe Clearances Using Foot-Worn Inertial Sensors. Proceedings of the 2019 12th International Congress on Image and Signal Processing, BioMedical Engineering and Informatics, CISP-BMEI.

[56] Sabatini A.M. (2011). Estimating Three-Dimensional Orientation of Human Body Parts by Inertial/Magnetic Sensing. Sensors.

[57] Pérez-Ibarra J.C., Williams H., Siqueira A.A.G., Krebs H.I. Real-Time Identification of Impaired Gait Phases Using a Single Foot-Mounted Inertial Sensor: Review and Feasibility Study. Proceedings of the 2018 7th IEEE International Conference on Biomedical Robotics and Biomechatronics (Biorob).

[58] Camomilla V., Bergamini E., Fantozzi S., Vannozzi G. (2018). Trends Supporting the In-Field Use of Wearable Inertial Sensors for Sport Performance Evaluation: A Systematic Review. Sensors.

[59] Wang L., Sun Y., Li Q., Liu T., Yi J. (2021). IMU-Based Gait Normalcy Index Calculation for Clinical Evaluation of Impaired Gait. IEEE J. Biomed. Health Inform..

[60] Manupibul U., Tanthuwapathom R., Jarumethitanont W., Kaimuk P., Limroongreungrat W., Charoensuk W. (2023). Integration of force and IMU sensors for developing low-cost portable gait measurement system in lower extremities. Sci. Rep..

[61] Yu G., Jang Y.J., Kim J., Kim J.H., Kim H.Y., Kim K., Panday S.B. (2016). Potential of IMU Sensors in Performance Analysis of Professional Alpine Skiers. Sensors.

[62] Gujarathi T., Bhole K. Gait Analysis Using Imu Sensor. Proceedings of the 2019 10th International Conference on Computing, Communication and Networking Technologies (ICCCNT).

[63] Liu T., Inoue Y., Shibata K. (2009). Development of a wearable sensor system for quantitative gait analysis. Measurement.

[64] Zhao H., Wang Z., Qiu S., Shen Y., Wang J. IMU-based gait analysis for rehabilitation assessment of patients with gait disorders. Proceedings of the 2017 4th International Conference on Systems and Informatics (ICSAI).

[65] Lou Y., Wang R., Mai J., Wang N., Wang Q. (2019). IMU-Based Gait Phase Recognition for Stroke Survivors. Robotica.

[66] García-de-Villa S., Ruiz L.R., Neira G.G.V., Álvarez M.N., Huertas-Hoyas E., del-Ama A.J., Rodríguez-Sánchez M.C., Seco F., Jiménez A.R. (2024). Validation of an IMU-based Gait Analysis Method for Assessment of Fall Risk Against Traditional Methods. IEEE J. Biomed. Health Inform..

[67] Ferrari A., Ginis P., Hardegger M., Casamassima F., Rocchi L., Chiari L. (2015). A mobile Kalman-filter based solution for the real-time estimation of spatio-temporal gait parameters. IEEE Trans. Neural Syst. Rehabil. Eng..

[68] Liu X., Zhao C., Zheng B., Guo Q., Duan X., Wulamu A., Zhang D. (2021). Wearable devices for gait analysis in intelligent healthcare. Front. Comput. Sci..

[69] Findlow A., Goulermas J.Y., Nester C., Howard D., Kenney L.P.J. (2008). Predicting lower limb joint kinematics using wearable motion sensors. Gait Posture.

[70] Suzuki Y., Hahn M.E., Enomoto Y. (2022). Estimation of Foot Trajectory and Stride Length during Level Ground Running Using Foot-Mounted Inertial Measurement Units. Sensors.

[71] Gouwanda D., Gopalai A.A., Khoo B.H. (2016). A Low Cost Alternative to Monitor Human Gait Temporal Parameters–Wearable Wireless Gyroscope. IEEE Sens. J..

[72] Hao M., Chen K., Fu C. (2019). Smoother-Based 3-D Foot Trajectory Estimation Using Inertial Sensors. IEEE Trans. Biomed. Eng..

[73] Li F., Liu G., Liu J., Chen X., Ma X. (2016). 3D tracking via shoe sensing. Sensors.

[74] Guimarães V., Sousa I., Correia M.V. (2021). A Deep Learning Approach for Foot Trajectory Estimation in Gait Analysis Using Inertial Sensors. Sensors.

[75] Romijnders R., Warmerdam E., Hansen C., Schmidt G., Maetzler W. (2022). A Deep Learning Approach for Gait Event Detection from a Single Shank-Worn IMU: Validation in Healthy and Neurological Cohorts. Sensors.

[76] Gonçalves H.R., Moreira R., Rodrigues A., Minas G., Reis L.P., Santos C.P. (2018). Real-time tool for human gait detection from lower trunk acceleration. Trends and Advances in Information Systems and Technologies.

[77] Carbonaro N., Lorussi F., Tognetti A. (2016). Assessment of a smart sensing shoe for gait phase detection in level walking. Electronics.

[78] Pham C., Diep N.N., Phuong T.M. e-Shoes: Smart shoes for unobtrusive human activity recognition. Proceedings of the 2017 9th International Conference on Knowledge and Systems Engineering (KSE).

[79] Fulk G.D., Edgar S.R., Bierwirth R., Hart P., Lopez-Meyer P., Sazonov E. (2012). Identifying activity levels and steps of people with stroke using a novel shoe-based sensor. J. Neurol. Phys. Ther..

[80] Kyoungchul K., Joonbum B., Masayoshi T. Detection of abnormalities in a human gait using smart shoes. Proceedings of the SPIE Smart Structures and Materials + Nondestructive Evaluation and Health Monitoring.

[81] Eskofier B., Lee S., Baron M., Simon A., Martindale C., Gaßner H., Klucken J. (2017). An Overview of Smart Shoes in the Internet of Health Things: Gait and Mobility Assessment in Health Promotion and Disease Monitoring. Appl. Sci..

[82] Sprager S., Juric M.B. (2015). Inertial Sensor-Based Gait Recognition: A Review. Sensors.

[83] Kanzler C.M., Barth J., Rampp A., Schlarb H., Rott F., Klucken J., Eskofier B.M. Inertial sensor based and shoe size independent gait analysis including heel and toe clearance estimation. Proceedings of the 2015 37th Annual International Conference of the IEEE Engineering in Medicine and Biology Society (EMBC).

[84] Park S.K., Suh Y.S. (2010). A Zero Velocity Detection Algorithm Using Inertial Sensors for Pedestrian Navigation Systems. Sensors.

[85] Kitagawa N., Ogihara N. (2016). Estimation of foot trajectory during human walking by a wearable inertial measurement unit mounted to the foot. Gait Posture.

[86] Nguyen L.V., La H.M. (2016). Real-Time Human Foot Motion Localization Algorithm with Dynamic Speed. IEEE Trans. Hum.-Mach. Syst..

[87] Guimarães V., Sousa I., Correia M.V. (2021). Orientation-Invariant Spatio-Temporal Gait Analysis Using Foot-Worn Inertial Sensors. Sensors.

[88] Merat P., Harvey E.J., Mitsis G.D. A Miniature Multi-sensor Shoe-Mounted Platform for Accurate Positioning. Proceedings of the 2018 IEEE International Conference on Systems, Man, and Cybernetics, SMC.

[89] Caruso M., Sabatini A.M., Laidig D., Seel T., Knaflitz M., Della Croce U., Cereatti A. (2021). Analysis of the Accuracy of Ten Algorithms for Orientation Estimation Using Inertial and Magnetic Sensing under Optimal Conditions: One Size Does Not Fit All. Sensors.

[90] Valenti R.G., Dryanovski I., Xiao J. (2015). Keeping a Good Attitude: A Quaternion-Based Orientation Filter for IMUs and MARGs. Sensors.

[91] Zrenner M., Gradl S., Jensen U., Ullrich M., Eskofier B.M. (2018). Comparison of different algorithms for calculating velocity and stride length in running using inertial measurement units. Sensors.

[92] Alaqtash M., Sarkodie-Gyan T., Yu H., Fuentes O., Brower R., Abdelgawad A. Automatic classification of pathological gait patterns using ground reaction forces and machine learning algorithms. Proceedings of the 2011 Annual International Conference of the IEEE Engineering in Medicine and Biology Society.

[93] Shetty S., Rao Y.S. SVM based machine learning approach to identify Parkinson’s disease using gait analysis. Proceedings of the 2016 International Conference on Inventive Computation Technologies (ICICT).

[94] Slijepcevic D., Zeppelzauer M., Gorgas A., Schwab C., Schüller M., Baca A., Breiteneder C., Horsak B. (2018). Automatic Classification of Functional Gait Disorders. IEEE J. Biomed. Health Inform..

[95] Derlatka M., Ihnatouski M. (2010). Decision Tree Approach to Rules Extraction for Human Gait Analysis. Artificial Intelligence and Soft Computing.

[96] Santhiranayagam B.K., Lai D., Begg R. (2015). Support vector machines for young and older gait classification using inertial sensor kinematics at minimum toe clearance. EAI Endorsed Trans. Pervasive Health Technol..

[97] Martinez-Hernandez U., Mahmood I., Dehghani-Sanij A.A. (2018). Simultaneous Bayesian Recognition of Locomotion and Gait Phases with Wearable Sensors. IEEE Sens. J..

[98] Lee M., Roan M., Smith B., Lockhart T.E. (2009). Gait analysis to classify external load conditions using linear discriminant analysis. Hum. Mov. Sci..

[99] Derlatka M., Bogdan M. Ensemble kNN classifiers for human gait recognition based on ground reaction forces. Proceedings of the 2015 8th International Conference on Human System Interaction (HSI).

[100] Lempereur M., Rousseau F., Rémy-Néris O., Pons C., Houx L., Quellec G., Brochard S. (2020). A new deep learning-based method for the detection of gait events in children with gait disorders: Proof-of-concept and concurrent validity. J. Biomech..

[101] Su B., Smith C., Farewik E.G. (2020). Gait Phase Recognition Using Deep Convolutional Neural Network with Inertial Measurement Units. Biosensors.

[102] Prakash C., Kumar R., Mittal N. (2018). Recent developments in human gait research: Parameters, approaches, applications, machine learning techniques, datasets and challenges. Artif. Intell. Rev..

[103] Alfayeed S.M., Saini B.S. Human Gait Analysis Using Machine Learning: A Review. Proceedings of the 2021 International Conference on Computational Intelligence and Knowledge Economy (ICCIKE).

[104] Saboor A., Kask T., Kuusik A., Alam M.M., Moullec Y.L., Niazi I.K., Zoha A., Ahmad R. (2020). Latest Research Trends in Gait Analysis Using Wearable Sensors and Machine Learning: A Systematic Review. IEEE Access.

